# Remimazolam induced cytotoxicity mediated through multiple stress pathways and acted synergistically with tyrosine kinase inhibitors in hepatocellular carcinoma

**DOI:** 10.1080/13510002.2025.2475696

**Published:** 2025-03-07

**Authors:** Hsiu-Lung Fan, Jia-Lin Chen, Shu-Ting Liu, Jia-Tong Lee, Shih-Ming Huang, Zhi-Fu Wu, Hou-Chuan Lai

**Affiliations:** a Division of General Surgery, Department of Surgery, Tri-Service General Hospital, National Defense Medical Center, Taipei City, Taiwan, Republic of China; b Department of Anesthesiology, Tri-Service General Hospital and National Defense Medical Center, Taipei, Taiwan, Republic of China; c Department of Biochemistry, National Defense Medical Center, Taipei City, Taiwan, Republic of China; d Department of Anesthesiology, Kaohsiung Medical University Hospital, Kaohsiung Medical University, Kaohsiung City, Taiwan, Republic of China; e Department of Anesthesiology, Faculty of Medicine, College of Medicine, Kaohsiung Medical University, Kaohsiung City, Taiwan, Republic of China; f Center for Regional Anesthesia and Pain Medicine, Wan Fang Hospital, Taipei Medical University, Taipei City, Taiwan, Republic of China

**Keywords:** Cytotoxicity, remimazolam-based balanced anesthesia, reactive oxygen species, drug synergy, cell death, mitochondrial dysfunction, tyrosine kinase inhibitor, stress

## Abstract

**Objectives:**

The primary treatment for hepatocellular carcinoma (HCC) involves surgical removal of the primary tumor, but this creates a favorable environment for the proliferation and spread of residual and circulating cancer cells. The development of remimazolam-based balanced anesthesia is crucial for future antitumor applications. It is important to understand the mechanisms of cytotoxicity for HCC in detail.

**Methods:**

We performed cell viability analysis, western blotting analysis, reverse transcription–polymerase chain reaction analysis, and flow cytometry analysis in two HCC cell lines, HepG2 and Hep3B cells.

**Results:**

Our data demonstrated that remimazolam induced cytotoxicity by suppressing cell proliferation, inhibiting G1 phase progression, and affecting mitochondrial reactive oxygen species (ROS) levels, leading to apoptosis, DNA damage, cytosolic ROS elevation, lipid peroxidation, autophagy, mitochondrial depolarization, and endoplasmic reticulum stress. Inhibitors of apoptosis, autophagic cell death, and ferroptosis and a ROS scavenger failed to rescue cell death caused by remimazolam besylate. Our combination index revealed that remimazolam besylate has the potential to act as a sensitizer for targeted tyrosine kinase inhibitor therapy for HCC.

**Discussion:**

Our findings open up new possibilities for combinatory HCC therapy using remimazolam, leveraging its dual functional roles in surgery and drug therapy for liver cancers.

## GRAPHICAL ABSTRACT

**Figure:**
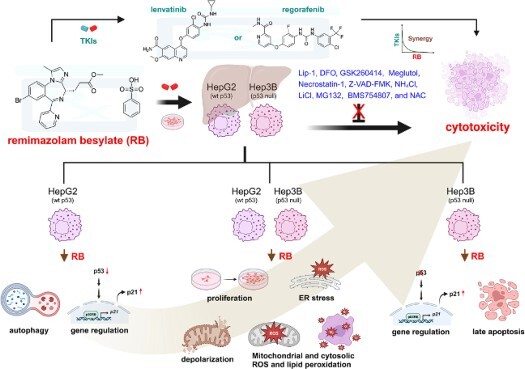

## Introduction

Remimazolam, an ultrashort-acting benzodiazepine analog with a favorable pharmacokinetic profile, has recently received approval for the induction and maintenance of general anesthesia [1]. It operates on γ-aminobutyric acid (GABA) type A (GABA-A) receptors and is rapidly converted into an inactive metabolite by liver-bound carboxylesterase, with a context-sensitive half-time of 7.5 minutes, making it suitable for use in the maintenance of general anesthesia. Among its numerous benefits, hemodynamic stability has been identified as the primary advantage. According to a recent systematic review and meta-analysis, remimazolam has been associated with a reduced risk of post-induction hypotension after anesthetic induction compared to propofol [2]. Kuang et al. reported that remimazolam (compared to propofol) may reduce the extent of short-term postoperative cognitive dysfunction, as measured by standard neuropsychological tests, optimize intraoperative hemodynamics, and improve oxygenation during one-lung ventilation [3]. In thoracoscopic laparoscopic radical esophagectomy, remimazolam demonstrated more stable hemodynamics and a lower incidence of adverse reactions compared to propofol [4]. Lee et al. reported that the administration of remimazolam with flumazenil might be a promising option for patients undergoing breast cancer surgery, leading to faster recovery and better sedation agitation scale scores than propofol during emergence from general anesthesia [5]. Regarding remimazolam-based balanced anesthesia, Shi et al. reported that remimazolam-0.6% sevoflurane is non-inferior to propofol-0.6% sevoflurane for general anesthesia during pituitary adenoma resection [6]. Zhang et al. demonstrated that remimazolam combined with propofol improved sedation and safety in hysteroscopy [7]. In painless gastroenteroscopy, Lu et al. showed that remimazolam combined with esketamine anesthesia had fewer side effects on patients’ circulatory and respiratory functions with fewer adverse effects compared to propofol anesthesia [8]. The development of remimazolam-based balanced anesthesia for various surgeries, including cancer surgery, is crucial for future applications.

Surgical removal of the primary tumor is a fundamental aspect of treatment for many types of cancer. However, it can also suppress the immune system and the host's tumor response, creating a favorable microenvironment for the proliferation and spread of residual and circulating cancer cells after the operation [9]. This not only ensures safety during perioperative changes, including surgical stress, blood transfusions, hypothermia, hyperglycemia, and postoperative pain, but also optimizes postoperative outcomes [10]. Therefore, it is crucial to carefully select anesthetic and analgesic agents during surgery to minimize the risk of promoting cancer recurrence [11]. Remimazolam has been shown to have benefits similar to those of dexmedetomidine in lowering the incidence of early postoperative cognitive dysfunction in aged patients after radical gastric cancer resection; this is possibly due to a reduced inflammatory response [12]. Yang also reported that remimazolam attenuated inflammation in bronchopneumonia through the inhibition of NOD-like receptor protein 3 inflammasome activity [13]. Remimazolam has been demonstrated to alleviate neuropathic pain, restrict pro-inflammatory factor production [14], and decrease cerebral ischemia/reperfusion injury [15]. Furthermore, it reduces oxidative stress and inflammation, offering protective advantages for the liver, lungs, and brain and supporting the maintenance of cognitive function in the brain [16]. Studies have shown that remimazolam inhibited glioma cell growth and induced apoptosis through the down-regulation of the NF-κB pathway [17] and inhibited the proliferation of colorectal cancer cells by reducing voltage-dependent anion channel expression [18].

Liver cancer ranks as the sixth most frequent cancer and the fourth leading cause of death globally, with 841,000 new cases and 782,000 deaths in 2018, accounting for 7% of all cancers [19–21]. Hepatocellular carcinoma (HCC) is the primary histological subtype of liver cancer, and its incidence rates increase with age. Immune checkpoint inhibitors, monoclonal antibodies, and tyrosine kinase inhibitors (TKIs) have shown more benefits than conventional therapies, such as chemotherapy, in the treatment of HCC [22]. The choice of therapy (chemotherapy, target therapy, or immunotherapy) or less effective therapy is determined by the type of primary malignant liver. However, the prognosis of late-stage HCC remains poor due to high recurrence rates, despite substantial advances in current therapeutic strategies. There is an urgent need for new treatments, such as a combination of the current systemic therapies. The antitumor mechanism of remimazolam in HCC has not been reported; however, it is possible that remimazolam could act as a sensitizer for the current therapies to overcome the challenge of clinical drug resistance.

The HepG2 and Hep3B cancer cell lines are among the most commonly used liver cancer cellular models in in vitro toxicity studies [23,24]. HepG2 possesses wild-type p53 and is HBV-negative and non-tumorigenic, while Hep3B is p53-deficient, HBV-positive, and tumorigenic. Many studies have indicated that the differences between HepG2 and Hep3B cell lines cannot be solely attributed to p53 or HBx. Furthermore, various disparities exist between the two cell lines, including variances in gene expression, drug responses, and associated signaling pathways, as documented in the literature [25–27]. In this study, we explored the cytotoxic effect of remimazolam besylate on the HepG2 and Hep3B cell lines. Additionally, we investigated the potential functional impact of remimazolam besylate on cell proliferation; cell cycle profiles; the oxidative response; autophagy; mitochondrial dysfunctions; the synergistic effects of TKIs, such as first-line lenvatinib and second-line regorafenib; and the differential responsiveness between HepG2 and Hep3B cells. Our findings may pave the way for a new approach to combination therapy for HCC using remimazolam, leveraging its dual functional roles in the surgical and drug therapy for liver cancers.

## Results

### The cytotoxic effects of remimazolam on two human hepatocellular carcinoma cells, HepG2 and Hep3B

**Figure 1.**
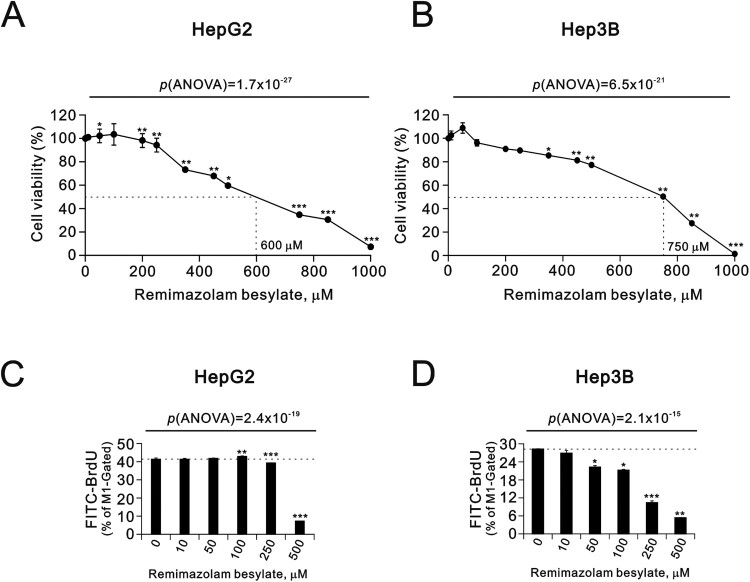
Effects of remimazolam besylate on cell proliferation in HepG2 and Hep3B cells. HepG2 and Hep3B cells were treated with the indicated concentrations of remimazolam besylate for 24 h. (A) Cell metabolic activity was measured using the MTT assay. (B) Cell proliferation was measured using the BrdU cell proliferation analysis. The results are representative of three independent experiments. Symbols and bars depict the mean ± SD. *p < 0.05, **p < 0.01, ***p < 0.001 (Student’s *t*-tests).

For in vitro toxicity studies, the HepG2 and Hep3B cancer cell lines are widely used as liver cancer cellular models [23,24]. In our study, we conducted a dose course of remimazolam besylate ranging from 0 to 1000 µM using the thiazolyl blue tetrazolium bromide (MTT) cytotoxicity assay for 24-hour treatment. Our data revealed that cell viability was suppressed around 100 µM in both cell lines, with individual IC_50_ concentrations of 600 µM for HepG2 and 750 µM for Hep3B, respectively (Figure 1(A,B). The significant suppression of cell viability by remimazolam besylate was found to be dose-dependent in both the HepG2 and the Hep3B cells (p(ANOVA) = 1.7 × 10^−27^ and p(ANOVA) = 6.5 × 10^−21^). We aimed to elucidate whether the cytotoxic effects of remimazolam besylate were mediated through cell proliferation and/or apoptosis. First, we examined the effect of remimazolam besylate on cell proliferation using the bromodeoxyuridine (BrdU) cell proliferation kit. We observed a significant inductive effect on the 100 µM remimazolam besylate-treated HepG2 cells, while significantly suppressive trends were observed in both the HepG2 and Hep3B cells (p(ANOVA) = 2.4 × 10^−19^ and p(ANOVA) = 2.1 × 10^−16^) (Figure 1(C,D)).

**Figure 2.**
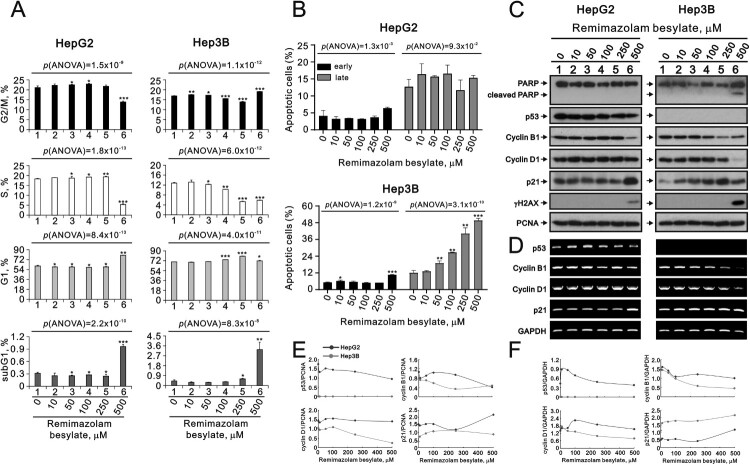
Effects of remimazolam besylate on cell cycle profile and apoptosis in HepG2 and Hep3B cells. HepG2 and Hep3B cells were treated with the indicated concentrations of remimazolam besylate for 24 h. (A) Cell cycle profiles were measured using the flow cytometry analysis with PI staining. (B) Cellular apoptosis was measured using the Annexin V apoptosis analysis with 7-AAD staining. Early apoptotic cells are PE Annexin V-positive and 7-AAD-negative, while late apoptotic cells are both PE Annexin V-positive and 7-AAD-positive. The results (A and B) are representative of three independent experiments. Symbols and bars depict the mean ± SD. *p < 0.05, **p < 0.01, ***p < 0.001 (Student’s *t*-tests). (C) Cell lysates were subjected to Western blot analysis and (D) RT-PCR analysis. PCNA was the protein loading control and *GAPDH* was the mRNA loading control. The protein and mRNA bands were quantified through pixel density scanning and evaluated using ImageJ, version 1.44a (http://imagej.nih.gov/ij/). (E) We plotted the ratios of protein to PCNA. (F) We plotted the mRNA/*GAPDH* ratios.

Subsequently, we assessed the effect of remimazolam besylate on the cell cycle profile using the PI dye. The populations of the S phase in the HepG2 and Hep3B cells were consistent with the findings of the BrdU data (p(ANOVA) = 1.8 × 10^−13^ and p(ANOVA) = 6.0 × 10^−12^) (Figure 2(A)). Our data also revealed cell cycle arrest in the G1 phase when the HepG2 and Hep3B cells were treated with remimazolam besylate, as evidenced by the increasing trend of G1 populations (p(ANOVA) = 8.4 × 10^−13^ and p(ANOVA) = 4.0 × 10^−11^) and the decreasing trend of cell proliferation. Additionally, the subG1 populations were significantly induced in both cells, with higher levels observed in the Hep3B cells than in the HepG2 cells. Furthermore, we verified the apoptotic cells using the Annexin V apoptosis assay kit. Our data demonstrated that remimazolam besylate significantly induced the early and late apoptotic Hep3B cells (p(ANOVA) = 1.2 × 10^−9^ and p(ANOVA) = 3.1 × 10^−10^), while it only significantly induced the early apoptotic HepG2 cells (p(ANOVA) = 1.3 × 10^−3^ and p(ANOVA) = 9.3 × 10^−2^) (Figure 2(B)). The cleaved PARP fragment was observed in the 500 µM remimazolam besylate-treated Hep3B cells in the Western blotting analysis (Figure 2(C,E)). Additionally, our Western blotting data showed that remimazolam besylate decreased the expression of cyclin B1 and cyclin D1 proteins and increased the expression of p21, a well-known p53 target gene, and γH2AX proteins in a dose-dependent manner. The status of p53 expression in the HepG2 cells, but not the Hep3B cells, served as a good control for cell identity. Our RT-PCR analytic data suggested that remimazolam besylate suppressed the expression of *p53* and *cyclin B1* mRNAs in the HepG2 cells and the expression of *cyclin B1* and *cyclin D1* mRNAs in the Hep3B cells (Figure 2(D,F)). The induction of *p21* mRNA by remimazolam besylate was observed in the HepG2 cells, which was consistent with its change in protein level, indicating regulation in a p53-independent manner for HepG2 and p53-deficient Hep3B cells. The regulation of *cyclin B1* and *cyclin D1* expressions might occur at the transcriptional stage.

### The effects of remimazolam on the oxidative stress, autophagy, and mitochondrial membrane potential in HepG2 and Hep3B cells

**Figure 3.**
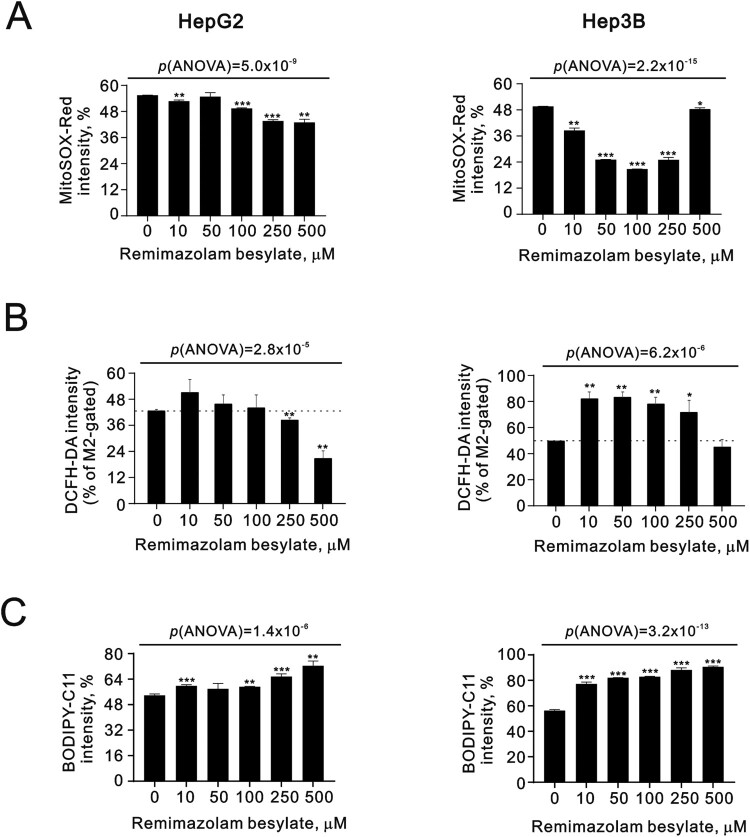
Effects of remimazolam besylate on ROS in HepG2 and Hep3B cells. HepG2 and Hep3B cells were treated with the indicated concentrations of remimazolam besylate for 24 h. They were then subjected to measurement of (A) MitoSOX intensity for mitochondrial ROS, (B) DCFH-DA intensity for cytosol ROS, and (C) BODIPY-C11 intensity for lipid peroxidation. The results are representative of three independent experiments. Symbols and bars depict the mean ± SD. *p < 0.05, **p < 0.01, ***p < 0.001 (Student’s *t*-tests).

Remimazolam has been shown to reduce oxidative stress and inflammation to some extent [16]. Therefore, we investigated the effect of remimazolam on reactive oxygen species (ROS) in the mitochondria and cytosol, which are two primary ROS generation sites, using MitoSOX and DCFH-DA, respectively. In the HepG2 cells, remimazolam besylate significantly suppressed the MitoSOX Red intensity in a dose-dependent manner. However, in the Hep3B cells, remimazolam besylate initially suppressed the MitoSOX Red intensity, but this rebounded to basal levels at 500 µM (Figure 3(A)) (p(ANOVA) = 5.0 × 10^−9^ and p(ANOVA) = 2.2 × 10^−15^). The levels of cytosolic ROS, as measured by DCFH-DA, initially increased at lower concentrations of remimazolam besylate and decreased at higher concentrations in both cell lines (Figure 3(B)). Imbalance in ROS generation rates can lead to oxidative stress and the production of free radicals that can damage DNA, proteins, and lipids. Lipid peroxidation can directly damage phospholipids and act as a cell death signal, inducing programmed cell death, such as apoptosis. We further measured the status of lipid peroxide with remimazolam besylate using BODIPY-C11 in the HepG2 and Hep3B cells. Our data showed that remimazolam besylate significantly induced lipid peroxidation in both cells (Figure 3(C)) (p(ANOVA) = 1.4 × 10^−6^ and p(ANOVA) = 3.2 × 10^−13^).

**Figure 4.**
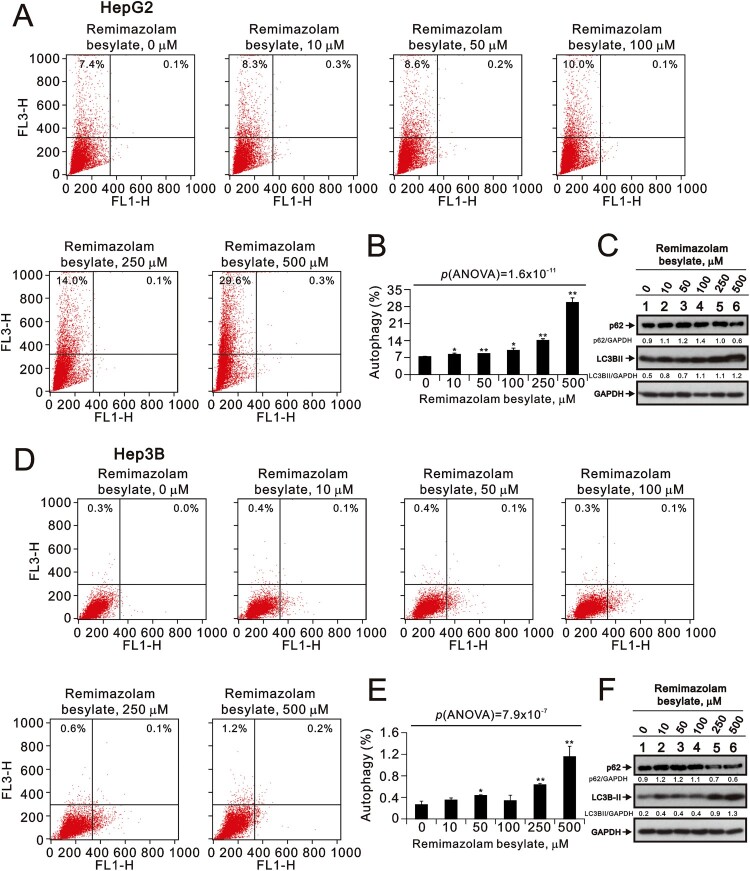
Effects of remimazolam besylate on autophagy in HepG2 and Hep3B cells. HepG2 and Hep3B cells were treated with the indicated concentrations of remimazolam besylate for 24 h. Acridine orange (1 µg/mL) staining was used to identify autophagic cells via FACS. (A and D) Acidic vesicular organelles were detected and quantified using acridine orange staining and measured using flow cytometry. (B and E) The intensity of the red fluorescence (y-axis, FL3-H) was proportional to the degree of acidity and the volume of acidic vesicular organelles, including autophagic vacuoles. The values refer to the percentages of cells with a significant proportion of acidic vesicular organelles. The results are representative of three independent experiments. Bars depict the means ± SDs. * *p* < 0.05; ** *p *< 0.01 (Student’s *t*-tests). (C and F) Cell lysates were subjected to Western blot analysis. GAPDH was the protein loading control. The protein bands were quantified through pixel density scanning and evaluated using ImageJ, version 1.44a. We listed the ratios of protein to GAPDH.

**Figure 5.**
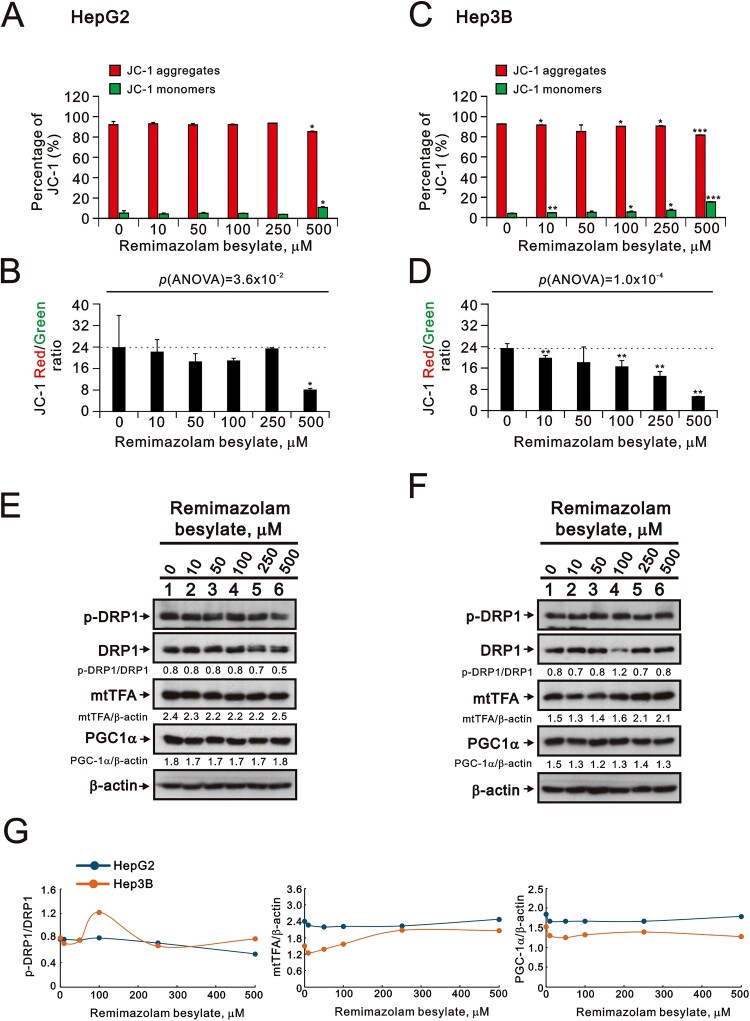
Effects of remimazolam besylate on mitochondrial membrane potential in HepG2 and Hep3B cells. HepG2 and Hep3B cells were treated with the indicated concentrations of remimazolam besylate for 24 h. Mitochondrial membrane potential was assessed using flow cytometry with JC-1 staining. (A and C) The percentages of red and green fluorescence intensities are plotted. (B and D) The red/green fluorescence intensity ratios were measured and are plotted. Bars depict the means ± SDs of three independent experiments. * *p* < 0.05; ** *p *< 0.01 (Student’s *t*-tests). (E and F) Cell lysates were subjected to Western blot analysis. β-actin was the protein loading control. The protein bands were quantified through pixel density scanning and evaluated using ImageJ, version 1.44a. (G) We plotted the ratios of protein to β-actin.

Autophagy serves to remove damaged mitochondria and oxidized proteins, supporting cell survival in most cases. Based on the increased levels of lipid peroxide induced by remimazolam besylate, we used acridine orange to measure the status of autophagy in the HepG2 and Hep3B cells. Remimazolam besylate significantly induced autophagy in a dose-dependent manner in the HepG2 cells; this was further verified by the autophagic biomarkers p62 (decreased) and LC3BII (increased) (Figure 4(A–C)) (p(ANOVA) = 1.6 × 10^−11^). Similar patterns were observed in the Hep3B cells, although the induced percentage of autophagic cells was lower than in the HepG2 cells (Figure 4(D–F)) (p(ANOVA) = 7.9 × 10^−7^). In addition to autophagy, the products of lipid peroxidation can adduct to specific mitochondrial proteins, driving cellular dysfunction. Therefore, we examined the effect of remimazolam besylate on mitochondrial membrane potential using the JC-1 dye. Our data showed that remimazolam besylate significantly decreased the red/green fluorescence intensity ratio of the HepG2 and Hep3B cells in a dose-dependent manner (Figure 5(A–D)) (p(ANOVA) = 3.6 × 10^−2^ and p(ANOVA) = 1.0 × 10^−4^). However, the populations of green fluorescence were relatively low, suggesting that the level of mitochondrial depolarization is not predominant for the function of remimazolam besylate in both cells. The Western blotting data showed that the mitochondrial biogenesis protein PGC-1α was not affected by remimazolam besylate in the HepG2 and Hep3B cells, but mtTFA was induced in the Hep3B cells (Figure 5(E–G)). The induction of the p-DRP1/DRP ratio was observed in the HepG2 cells.

### The effects of remimazolam on stress and signaling proteins in HepG2 and Hep3B cells

**Figure 6.**
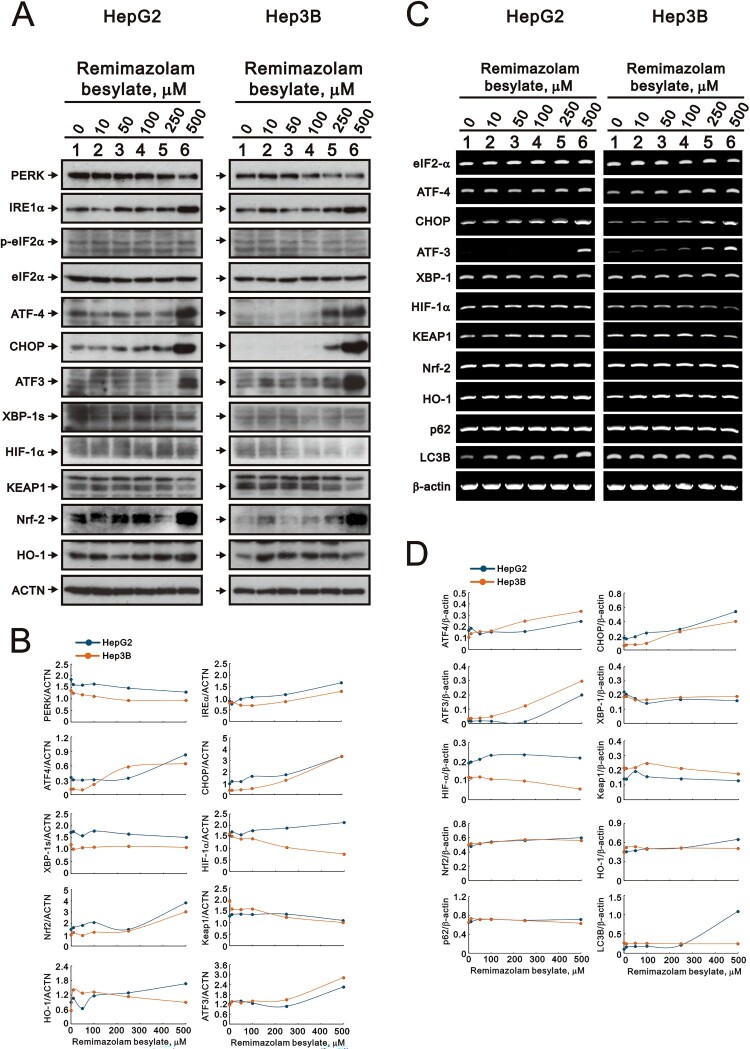
Effects of remimazolam besylate on target proteins and mRNAs in HepG2 and Hep3B cells. HepG2 and Hep3B cells were treated with the indicated concentrations of remimazolam besylate for 24 h. Cell lysates were subjected to (A) Western blot analysis and (C) RT-PCR analysis. ACTN was the protein loading control and *β-actin* was the mRNA loading control. The protein and mRNA bands were quantified through pixel density scanning and evaluated using ImageJ, version 1.44a (http://imagej.nih.gov/ij/). (B) We plotted the ratios of protein to ACTN. (D) We plotted the mRNA/*β-actin* ratios.

Our findings revealed that remimazolam besylate influenced the status of ROS, autophagy, and mitochondrial membrane potential in the HepG2 and Hep3B cells. Endoplasmic reticulum (ER) stress accumulates following the unfolding protein response (UPR) and is the primary cause of ROS and autophagy. A prominent characteristic of ER stress is associated with the up-regulation of the transcription factor CCAAT/enhancer-binding protein homologous protein (CHOP). In addition to CHOP, we examined target proteins and mRNAs related to UPR, ER stress, and ROS using Western blotting and RT-PCR analysis in HepG2 and Hep3B cells (Figure 6).

In the Western blotting analysis, we observed that remimazolam besylate increased the UPR proteins IRE-1α, ATF4, and ATF3; decreased the UPR proteins PERK and eIF2α; and had no effect on the XBP-1s protein and the ratio of p-eIF2α/eIF2α in the HepG2 and Hep3B cells (Figure 6(A,B)). Remimazolam besylate induced the expression of the CHOP (an ER stress biomarker) and Nrf2 (a ROS biomarker) proteins and decreased the expression of the Keap1 protein (Figure 6(A,B)). The expression levels of the *ATF4*, *ATF3*, and *CHOP* mRNAs were consistent with their proteins (Figure 6(C,D)). However, HO-1, a target gene of Nrf2, protein and mRNA were increased by remimazolam in the HepG2 cells, but not in the Hep3B cells. Autophagic *LC3B* mRNA was increased in the HepG2 cells, and *p62* and *LC3B* mRNAs were decreased in the Hep3B cells (Figure 6(C,D)). HIF-1α protein and mRNA were decreased by remimazolam besylate in the Hep3B cells, but not in the HepG2 cells.

**Figure 7.**
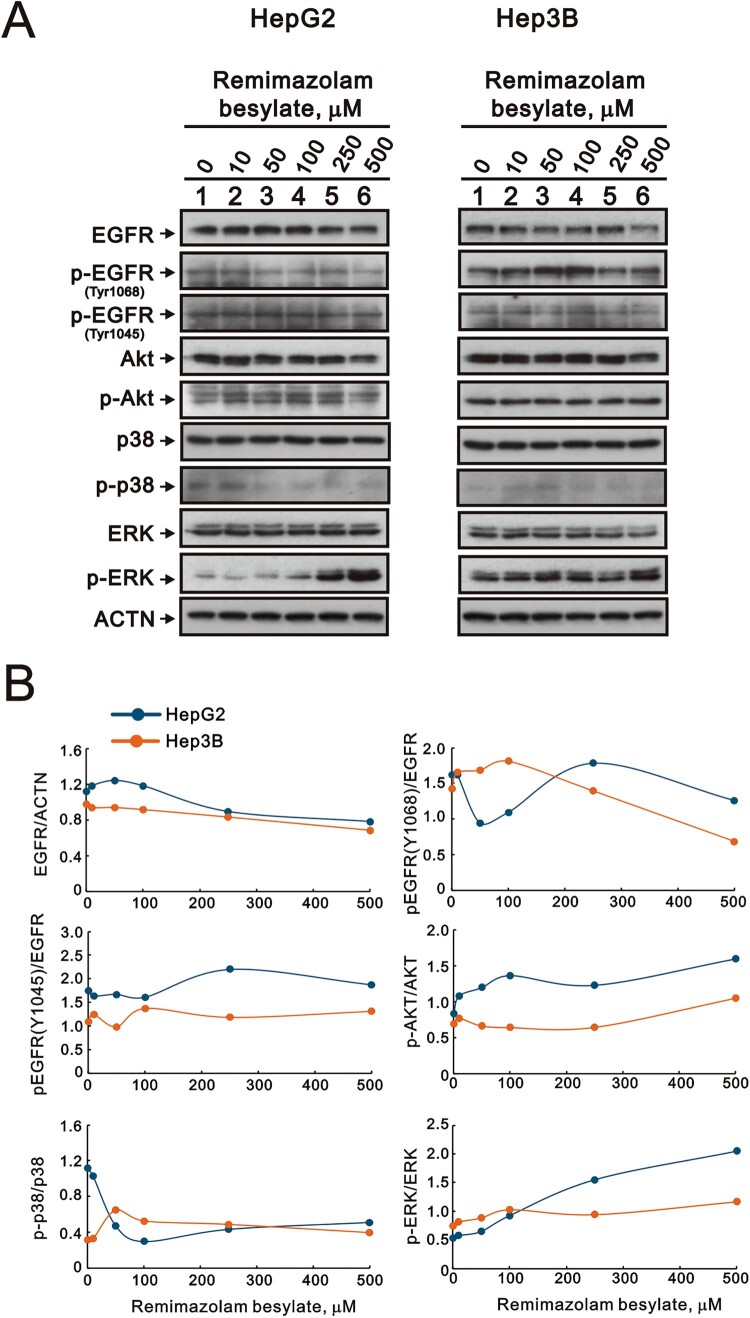
Effects of remimazolam besylate on the signaling pathway in HepG2 and Hep3B cells. (A) HepG2 and Hep3B cells were treated with the indicated concentrations of remimazolam besylate for 24 h. Cell lysates were subjected to Western blot analysis. ACTN was the protein loading control. The protein bands were quantified through pixel density scanning and evaluated using ImageJ, version 1.44a. (B) We plotted the ratios of protein to ACTN and phosphorylated protein to total protein.

Remimazolam acts on the GABA-A receptor. The phosphorylation of EGFR on specific sites (Y1045 and Y1068) creates binding sites for Grb2, leading to the activation of the MAPK/ERK cascade, and a binding site for Gab1, which recruits the p85 subunit of phosphatidylinositol 3-kinase (PI 3-kinase), leading to AKT activation. Hence, we examined which signaling pathways, including EGFR, AKT, p38, and ERK, were activated by remimazolam besylate in the HepG2 and Hep3B cells. Our Western blotting data revealed that remimazolam besylate elevated the ratios of p-Akt/Akt and p-ERK/ERK in both HepG2 and Hep3B cells (Figure 7(A,B)). Additionally, remimazolam besylate decreased the overall expression of total EGFR in both cell types and lowered the pEGFR/EGFR ratios. Notably, an increase in the pEGFR(Y1068)/EGFR ratio was specifically observed below 250 μM remimazolam besylate in Hep3B cells. Furthermore, remimazolam besylate reduced the p-p38/p38 ratio in HepG2 cells while increasing the p-p38/p38 ratio in Hep3B cells.

### The combination index of tyrosine kinase inhibitor and remimazolam was analyzed in HepG2 and Hep3B cells

**Figure 8.**
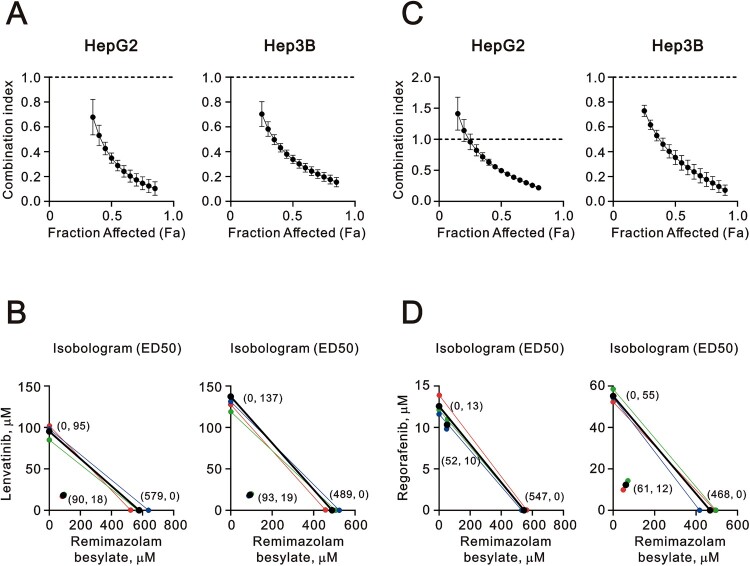
Combination index of remimazolam besylate with TKIs lenvatinib and regorafenib in HepG2 and Hep3B cells. HepG2 and Hep3B cells were treated with remimazolam besylate dose: 0, 3.125, 6.25, 12.5, 25, 50, 100, 200, 400, 800 µM combined with (A and B) lenvatinib dose: 0, 2.5, 5, 10, 20, 40, 80, 160 μM or (C and D) regorafenib dose: 0, 2.5, 5, 10, 20, 40, 80, 160 μM. Cell viability was measured using the MTT method. The combination index of remimazolam besylate plus (B) lenvatinib or (D) regorafenib. Isobolograms (ED_50_) of remimazolam plus lenvatinib or regorafenib were calculated from three independent experiments (red, blue, and green dots and lines) using CalcuSyn software (black dots and lines).

Sorafenib and lenvatinib are recognized as the most effective TKIs and first-line single-drug therapies. Additionally, cabozantinib, ramucirumab, and regorafenib have shown improved survival benefits in second-line therapies following first-line treatment with sorafenib [22]. Numerous phase III trials have been conducted to investigate the effectiveness of combination therapy. We aimed to elucidate the potential cytotoxicity mechanism of the combination therapy of remimazolam with TKIs, including lenvatinib and regorafenib, using combination index analysis (Figure 8). The combination index (CI) between remimazolam besylate and lenvatinib or regorafenib was found to be below 1, suggesting that remimazolam besylate acted synergistically with lenvatinib or regorafenib in the HepG2 and Hep3B cells (Figure 8(A,C)). Our data revealed that the EC_50_ of remimazolam besylate was 579 or 547 µM in the HepG2 cells and 489 or 468 µM in the Hep3B cells; the EC_50_ of lenvatinib was 95 µM in the HepG2 cells and 137 µM in the Hep3B cells; and the EC_50_ of regorafenib was 13 µM in the HepG2 cells and 55 µM in the Hep3B cells (Figure 8(B,D)). Furthermore, the combination of remimazolam besylate reduced the EC_50_ of lenvatinib from 95 µM to 18 µM in the HepG2 cells and from 137 µM to 19 µM in the Hep3B cells, at 90 and 93 µM of remimazolam besylate, respectively. Similarly, the EC_50_ of regorafenib was reduced from 13 µM to 10 µM in the HepG2 cells and from 55 µM to 12 µM in the Hep3B cells, at 52 and 61 µM of remimazolam besylate, respectively (Figure 8(B,D)). Based on our results, remimazolam may serve as a potential sensitizer for VEGFR-targeted TKIs to overcome the current challenge of clinical resistance.

### The differential responsiveness of various inhibitors in HepG2 and Hep3B cells

**Figure 9.**
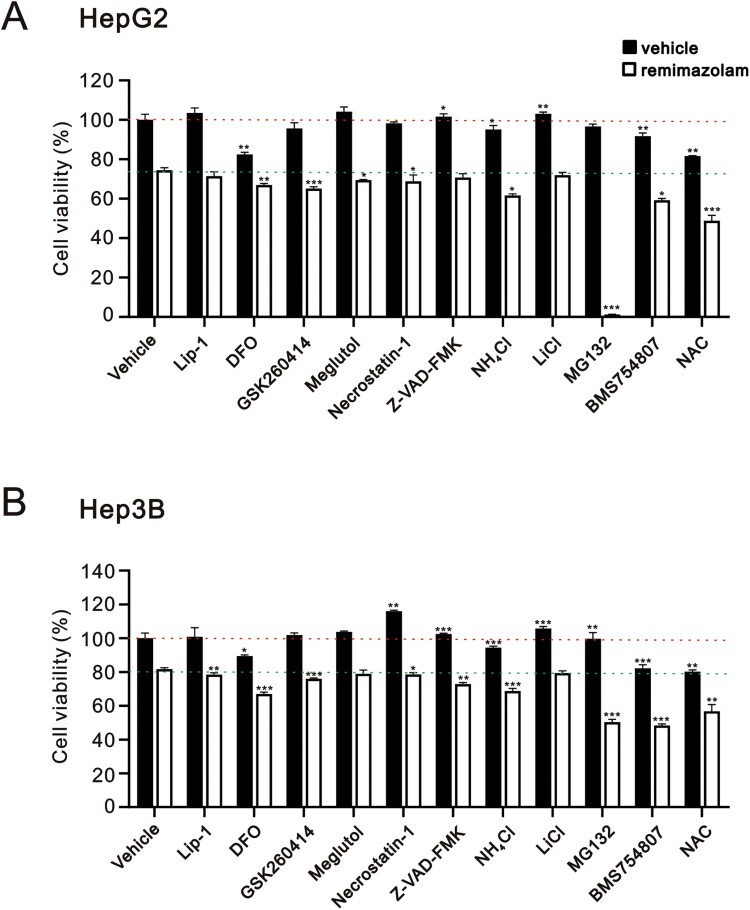
The ability of various cell death inhibitors to rescue remimazolam besylate-mediated cytotoxicity in HepG2 and Hep3B cells. (A) HepG2 and (B) Hep3B cells were pre-treated for 1 h with indicated inhibitors, including 2 µM Lip-1, 100 µM DFO, 4 µM GSK260414, 5 µM Meglutol, 40 µM Necrostatin-1, 40 µM Z-VAD-FMK, 10 mM NH_4_Cl, 10 mM LiCl, 0.1 µM MG132, 0.1 µM BMS754807, and 10 mM NAC, and combined with vehicle or 400 μM remimazolam besylate for 24 h. Cell viability was measured using the MTT method. Bars depict the means ± SDs of three independent experiments. * *p* < 0.05; ** *p *< 0.01; *** *p *< 0.001 (Student’s *t*-tests). The dashed red line is the level for the vehicle control, and the dashed green line is the level for the remimazolam besylate alone.

Our findings demonstrated that remimazolam besylate induced cytotoxicity through the induction of apoptosis, DNA damage, cytosolic ROS levels, lipid peroxidation, autophagy, mitochondrial depolarization, and ER stress in HepG2 and Hep3B cells. The observed cytotoxic effects exhibited some differences between the two cell lines. Therefore, we applied various inhibitors, including lipid peroxide inhibitor liprosatin-1 (Lip-1), iron chelator deferoxamine (DFO), ER stress PERK inhibitor GSK620414, antilipemic agent meglutol, necroptosis inhibitor necrostatin-1, apoptosis inhibitor Z-VAD-FMK, lysosome inhibitor NH_4_Cl, glycogen synthase kinase-3 inhibitor LiCl, cell-permeable proteasome inhibitor MG132, insulin receptor family kinase inhibitor BMS754807, and ROS scavenger N-acetyl cysteine (NAC), to examine their individual cytotoxic effects on HepG2 and Hep3B cells and their ability to rescue the cytotoxic effect of remimazolam besylate (Figure 9). Our data revealed that Z-VAD-FMK and LiCl significantly enhanced the cell viability of HepG2 cells, while Necrostatin-1, Z-VAD-FMK, LiCl, and MG132 significantly enhanced the cell viability of Hep3B cells (Figure 9(A,B)). Conversely, DFO, NH_4_Cl, BMS754807, and NAC suppressed the cell viability of both cell lines.

None of the tested inhibitors rescued the cytotoxic effect of remimazolam besylate on HepG2 and Hep3B cells (Figure 9(A,B)). Furthermore, DFO, GSK620414, Necrostatin-1, NH_4_Cl, MG132, BMS754807, and NAC significantly potentiated the cytotoxic effect of remimazolam besylate on both cell lines (Figure 9(A,B)). Additionally, meglutol significantly potentiated the cytotoxic effect of remimazolam besylate on HepG2 cells, while Lip-1 and Z-VAD-FMK significantly potentiated the cytotoxic effect of remimazolam besylate on Hep3B cells.

## Discussion

In this study, we demonstrated that remimazolam besylate may induce cytotoxicity by suppressing cell proliferation, G1 phase progression, and mitochondrial ROS levels, while also inducing apoptosis, DNA damage, cytosolic ROS levels, lipid peroxidation, autophagy, mitochondrial depolarization, and ER stress in HepG2 and Hep3B cells. Our rescue data suggest that the drug responsiveness of remimazolam besylate or the inhibitors differs between HepG2 and Hep3B cells, and the mechanism of cytotoxicity induced by remimazolam besylate may be mediated through multiple pathways in both cell lines. Apoptosis, autophagic cell death, and lipid peroxide-induced ferroptosis may be primary causes of the cell death induced by remimazolam besylate in HepG2 and Hep3B cells. Z-VAD-FMK, NH_4_Cl, Lip-1, DFO, and meglutol, which inhibit apoptosis, autophagic cell death, and ferroptosis, respectively, all failed to rescue the respective cell death induced by remimazolam besylate. Furthermore, our data revealed that remimazolam besylate may result in G1 phase arrest based on the suppression of cell proliferation in HepG2 and Hep3B cells. Therefore, the significant induction of cytotoxicity by remimazolam besylate may result from the suppression of cell proliferation and the induction of cell death (apoptosis, autophagic cell death, or lipid peroxide-induced ferroptosis) in both cell lines. Our Western blotting and RT-PCR analysis showed that the induction of G1 phase arrest mediated through the suppression of cyclin D1 protein and the induction of p21 protein may occur in a p53-independent manner in p53-deficient Hep3B cells. In HepG2 cells, remimazolam besylate may suppress p53 and cyclin D1 proteins and induce p21 protein and mRNA mediated through a p53-independent manner for G1 phase arrest. Additionally, the proteasome inhibitor MG132 potentiated the remimazolam besylate-induced cytotoxic effect, indicating that protein stability plays an important role in remimazolam besylate-induced cytotoxicity in both cell lines. Our combination index revealed that remimazolam besylate has the potential to act as a sensitizer for targeted tyrosine kinase inhibitor therapy for HCC.

ROS-induced cell death can trigger caspase-dependent apoptosis and caspase-independent cell death pathways, such as necroptosis, ferroptosis, and autophagic cell death [28,29]. Polyunsaturated fatty acids are particularly susceptible to ROS damage, in a process known as lipid peroxidation [30]. However, the products of lipid peroxidation can form adducts with specific mitochondrial and autophagy-related proteins, leading to cellular dysfunction in an autophagic cell death pathway. Autophagy plays a protective role by eliminating ROS to maintain mitochondrial integrity and prevent apoptosis. Under normal conditions, ROS-induced autophagy reduces the damage caused by oxidative stress to protect cells. However, excessive autophagy induced by ROS can also lead to autophagic cell death under certain circumstances. In this study, our findings demonstrated that remimazolam besylate induced lipid peroxidation, disrupting mitochondrial and cytosolic ROS homeostasis in HepG2 and Hep3B cells. Our data revealed that, compared with the cytotoxic effects of remimazolam besylate on both cell types, ferroptosis and autophagic cell death might be the cause of cell death induced by remimazolam besylate in HepG2 cells, while ferroptosis and apoptosis might be the cause of cell death induced by remimazolam besylate in Hep3B cells. The inhibitors for apoptosis, autophagy, and ferroptosis, as well as ROS scavengers, failed to rescue the cytotoxicity induced by remimazolam besylate. In addition to the ROS scavenger, the main intracellular sensor that monitors ROS is KEAP1 [31]. Prolonged or substantial oxidative stress, however, may not induce a sufficient expression of cytoprotective proteins and can result in cell damage and/or cell death. In comparison, our Western blotting data also demonstrated that remimazolam induced the Nrf2 protein, but not its downstream *HO-1* mRNA and protein, suggesting that elucidating the detailed regulatory mechanism of Keap1-Nrf2 in response to substantial oxidative stress in HepG2 and Hep3B cells is an important task.

The activation of voltage-gated Ca^2+^ channels in GH4 pituitary cells can prevent Gl phase cells from entering the S phase and can also promote the progression of G2 cells through mitosis [32]. At least two phases of the cell cycle, the transition of cells in the Gl phase into the S phase and progression through the M phase, are highly Ca^2+^-dependent. Depolarizing GABA actions lead to a decrease in both DNA synthesis and the number of BrdU-labeled cells in the subventricular zone in the rat embryonic neocortex [33]. In this study, remimazolam may act similarly to GABA through GABA-A receptors for the activation of voltage-gated Ca^2+^ channels to decrease cell proliferation and block Gl phase cells from entering the S phase in HepG2 and Hep3B cells. However, further verification is needed to determine whether this function is mediated through the Ca^2+^-dependent pathway. The activation of the PI3 K/Akt and Ras/Raf/MEK/MAPK signaling pathways downstream from the EGFR has been proposed as an important signaling mechanism [34]. The six autophosphorylation sites in the COOH-terminal tail of EGFR are tyrosine residues 992, 1045, 1068, 1086, 1148, and 1173. Once phosphorylated, these residues serve as docking sites and facilitate a connection between the external stimulation and specific internal signal transduction pathways. Our data may link the GABA-A receptor to EGFR pathways for the activation of different signaling pathways by remimazolam besylate, depending on the differential expression and function of GABA-A receptor subtypes in HepG2 and Hep3B cells.

Currently, a balanced anesthesia strategy is adopted in clinical operations, leveraging the advantages of multiple anesthetic drugs and administering smaller doses to avoid the side effects caused by larger doses of a single anesthetic drug, such as sevoflurane-propofol anesthesia or remimazolam-propofol anesthesia [35,36]. Midazolam, a derivative of BZD, is extensively used for procedural sedation and the induction of general anesthesia in clinical practice [37]. Midazolam/fentanyl sedation was associated with an increased periprocedural perception of pain and lower local tumor progression-free survival compared to propofol and general anesthesia in patients with hepatic malignancies [38]. Midazolam exhibited antitumor properties, enhanced the efficiency of Anti-PD-1 immunotherapy, inhibited proliferation, and accelerated the apoptosis of HCC cells [37,39]. In this study, our combination index revealed that remimazolam besylate potentially served as a sensitizer of TKI target therapy for liver cancer. The lower 10-fold cytotoxic dosage of remimazolam besylate in combination therapy, compared to single therapy, may help minimize the detrimental effects on healthy cells. When compared to the current dosages used for general anesthetic applications lasting 1–8 hours, combining remimazolam with AKIs is anticipated to provide specific advantages for targeting tumor cells while protecting normal cells. Therefore, the development of a remimazolam-based balanced anesthesia to avoid the suppression of the immune system and metastasis during liver cancer surgery is important for future antitumor applications.

## Methods and materials

### Cell culture and reagents

The HepG2 (CVCL_0027) and Hep3B (CVCL_0326) HCC cell lines were obtained from the American Type Culture Collection (ATCC; Manassas, VA, USA). The cells were cultivated in Dulbecco’s modified Eagle’s medium (DMEM) supplemented with 10% (v/v) fetal bovine serum (FBS) and 1% (w/v) penicillin – streptomycin (Thermo Fisher Scientific, Waltham, MA, USA). The cell lines were regularly tested for mycoplasma infections using a Mycoplasma Detection Kit (InvivoGen, San Diego, CA, USA) and were regularly changed by thawing stock cell lines, as previously described [40]. 2′,7-dichlorofluorescein diacetate (DCFH-DA), lenvatinib, propidium iodide (PI), regorafenib, remimazolam besylate, and MTT were purchased from Sigma Aldrich (St. Louis, MO, USA). Human HCC cell lines were handled using good cell culture practice in this study. No human or animal ethical approval was obtained.

### Cell survival analysis and calculated combination index

The cells were plated in 96-well culture plates and treated with the indicated concentrations of remimazolam besylate. The cells were then incubated with MTT solution for 1 h at 37°C. Dimethyl sulfoxide (DMSO; 200 μl) was then added, and the absorbances at 570 and 650 nm were measured using an ELISA plate reader (Multiskan EX, Thermo, MA, USA). The control group containing cells cultured in medium only was defined as representing 100% cell survival. The combination index (CI) was calculated using CalcuSyn (Biosoft, Cambridge, UK) to generate the isobologram, as previously described [41]. In general, CI <1 indicates a synergistic combination effect and CI >1 indicates an additive combination effect [42].

### Fluorescence-activated cell sorting (FACS), cell proliferation, cell cycle Profiling, apoptosis, ROS, lipid peroxidation, acidic vesicular organelles, and mitochondrial membrane potential analyses

The cell proliferation was assessed using immunofluorescent staining with incorporated BrdU (BD Pharmingen™ BrdU Flow Kit) and flow cytometry, according to the manufacturer’s instructions. Briefly, the cells were seeded in 6-well culture plates and treated with various concentrations of remimazolam besylate for 24 h. After incubation, the cells were stained with BrdU, harvested, washed with PBS, and then fixed and permeabilized before being stained with BrdU with fluorescent antibodies. The cells were resuspended in staining buffer, and an FITC-BrdU fluorescence analysis was performed using a FACSCalibur flow cytometer and Cell Quest Pro software (BD Biosciences, CA, USA), as previously described [41].

The cell cycle profiles were measured according to their cellular DNA content using FACS. The cells were fixed in 70% (v/v) ice-cold ethanol, stored at −30 °C overnight, washed two times with ice-cold PBS supplemented with 1% (v/v) FBS, and then stained with PI solution (5 μg/mL PI in PBS, 0.5% (v/v) Triton X-100, and 0.5 μg/mL RNase A) for 30 min at 37 °C in the dark. Then, they were evaluated using a FACSCalibur flow cytometer and Cell Quest Pro software (BD Biosciences, Franklin Lakes, NJ, USA), as previously described [40].

The early and late stages of the apoptotic cells were evaluated using a fluorescein Phycoerythrin (PE)-Annexin V Apoptosis Detection Kit (BD Biosciences). In accordance with the manufacturer’s protocol, the cells were stained with PE-Annexin V as well as 7-AAD to determine the effects of the indicated concentrations of remimazolam besylate on early apoptosis (PE-Annexin V positive and 7-AAD negative) and late apoptosis (PE-Annexin V positive and 7-AAD positive), as previously described [41].

The intracellular ROS levels were determined using the fluorescent marker DCFH-DA. The cells were exposed to various concentrations of remimazolam besylate for 24 h, stained with DCFH-DA (10 μM) for 40 min at 37 °C, and then harvested. After washing the cells once with PBS, the fluorescence was analyzed on channel FL-1 of the FACSCalibur flow cytometer using Cell Quest Pro software (BD Biosciences, Franklin Lakes, NJ, USA). The cell volume gating strategy involved forward scatter height (FSC-H) and side scatter height (SSC-H), and the median fluorescence intensity of the vehicle was used as the starting point for M2 gating, as previously described [40]. Mitochondrial superoxide production is an important source of reactive oxygen species in cells that may cause disease. The MitoSOX^TM^ Red (Invitrogen, Thermo Fisher Scientific, M36008) mitochondrial superoxide indicator is a fluorogenic dye for the highly selective detection of superoxide in the mitochondria of live cells. Once in the mitochondria, MitoSOX^TM^ Red reagent is oxidized by the superoxide and exhibits red fluorescence. Briefly, the cells were seeded in 6-well culture plates and treated with indicated concentrations of remimazolam besylate for 24 h. After incubation, the cells were harvested and stained with 5 μM MitoSOX^TM^ Red at 37 °C for 20 min, washed once with PBS, and resuspended in PBS. The MitoSOX^TM^ Red fluorescence was then analyzed using flow cytometry (FACSCalibur, BD Biosciences); the fluorescence intensity was analyzed on the FL-2 channel of the FACSCalibur flow cytometer using Cell Quest Pro software, version 6.1 (BD Biosciences), as previously described [43].

The cell lipid peroxidation status was determined using the BODIPY^TM^ 581/591 C11 (Invitrogen^TM^) fluorescent dye. The cells were exposed to the indicated concentrations of remimazolam besylate for 24 h and then harvested. The cells were stained with BODIPY-C11 581/591 (10 μM) for 30 min at 37 °C; after washing the cells once with PBS, the fluorescence intensity was analyzed on channel FL-1 of the FACSCalibur flow cytometer using Cell Quest Pro software (BD Biosciences, Franklin Lakes, NJ, USA). In the reduced state, the BODIPY-C11 excitation and emission maxima were 581/591 nm; after oxidation, the fluorescence shifted the excitation and emission to 488/510 nm.

The acidic compartments of the cells were detected using acridine orange (Sigma, Cat. No. A8097) staining and measured with flow cytometry. As the protonated form of acridine orange accumulates inside acidic vesicles, it is a marker for the final steps of the autophagy process. Briefly, the cells were treated with the indicated concentrations of remimazolam besylate for 24 h, stained with acridine orange (1 μg/ml) for 20 min at 37°C, and then trypsinized for harvesting. Afterwards, the cells were washed once with PBS, resuspended in 400 μl of PBS, and then analyzed via flow cytometry (FACSCalibur, BD, Biosciences). The excitation wavelength was 488 nm, and the fluorescence at 510–530 nm (green fluorescence, FL1) and 650 nm (red fluorescence, FL3) was detected. The data were analyzed using the CellQuest™ software program. The percentage of autophagic cells was calculated based on the number of cells present in the upper-left and upper-right quadrants, as previously described [43].

Mitochondrial depolarization was measured as a function of a decrease in the red/green fluorescence intensity ratio. All the dead and viable cells were harvested, washed with PBS, and incubated with 1× binding buffer containing the MMP-sensitive fluorescent dye JC – 1 for 30 min at 37 °C in the dark. After washing the cells once with PBS, JC-1 fluorescence was analyzed on channels FL-1 and FL-2 of the FACSCalibur flow cytometer using Cell Quest Pro software (BD Biosciences, Franklin Lakes, NJ, USA) to detect monomer (green fluorescence) and aggregate (red fluorescence) forms of the dye, respectively, as previously described [40].

### Western blotting analysis

The HepG2 and Hep3B cells were lysed in radioimmunoprecipitation assay buffer (100 mM Tris-HCl (pH 8.0), 150 mM NaCl, 0.1% (w/v) SDS, and 1% (v/v) Triton X-100) at 4 °C. The proteins in the resultant lysates were separated using sodium dodecyl sulfate–polyacrylamide gel electrophoresis and analyzed using immunoblotting with antibodies against α-sactinin (ACTN) (H-2, 1:5000 v/v), Nrf2 (H-6, 1:1000 v/v), p53 (DO-1, 1:2000v/v), p62 (D-3, 1:1000 v/v), Cyclin B1 (GNS1, 1:1000 v/v), PCNA (F-2, 1:5000 v/v), GAPDH (0411, 1:2000v/v), mtTFA (C-9, 1:1000 v/v), DRP1 (6Z-82, 1:1000 v/v), α-actin (C-4 1:5000 v/v), and PGC1α(D-5, 1:1000 v/v), XBP-1s (F-4, 1:1000 v/v) (Santa Cruz Biotechnology, Santa Cruz, CA, USA); cleaved poly-ADP-ribose polymerase (PARP) (9546, 1:1000 v/v), LC3B (2775, 1:1000 v/v), p-DRP1 (3455, 1:1000 v/v), PERK (3192, 1:1000 v/v), p-eIF2α (9721, 1:1000 v/v), eIF2α (9722, 1:1000 v/v), ATF4 (11815, 1:1000 v/v), CHOP (2895, 1:1000 v/v), ATF3 (33593, 1:1000 v/v), HIF1α (14179, 1:1000 v/v), EGFR (4267, 1:1000 v/v), p-EGFR (Tyr1068) (3777, 1:1000 v/v), p-EGFR (Tyr1045) (2237, 1:1000 v/v), Akt (4691, 1:2000v/v), p-Akt (9271, 1:1000 v/v), p38 (9212, 1:2000v/v), p-p38 (9211, 1:1000 v/v), ERK (4695, 1:2000v/v), and p-ERK (4370, 1:1000 v/v) (Cell Signaling, Danvers, MA, USA); cyclin D1 (ab134175, 1:2000v/v), γH2AX (ab81299, 1:1000 v/v), p21 (ab109520, 1:1000 v/v), and IRE1α (ab48187, 1:1000 v/v) (Abcam, Cambridge, UK); HO-1 (heme oxygenase-1, 1:2000v/v) (ADI-SPA-895-F) (Enzo Life Sciences, Farmingdale, NY, USA); and KEAP1 (60027-1-Ig, 1:1000 v/v) (Proteintech). The blots were subsequently incubated with HRP-conjugated secondary antibodies (anti-mouse IgG, AP192P, 1:5000 v/v, and anti-rabbit IgG, AP132P, 1:5000 v/v, Merck-Millipore). The immunoreactive proteins were detected using the ECL^TM^ Western Blotting Detection Reagent and Amersham HyperfilmTM ECL (GE Healthcare, USA), as previously described [41].

### Reverse transcription–polymerase chain reaction (RT-PCR)

The total RNAs were isolated from the HepG2 and Hep3B cells using TRIzol reagent (Invitrogen). Reverse transcription for first-strand cDNA synthesis was carried out using MMLV reverse transcriptase (Epicentre Biotechnologies, USA) with 1 μg of total RNA for 60 min at 37°C. The PCRs were run on a Veriti Thermal Cycler (Applied Biosystems, USA), as previously described [41]. The PCR primers are listed in Table 1.

**Table 1.** The PCR primers were used in this study.

Primer Name	Accession number	Sequence (5′→3′)
*β-actin*	NM_001101.3	Forward: 5′-GTGGGGCGCCCCAGGCACCA-3′
		Reverse: 5′-CTCCTTAATGTCACGCACGATTTC-3′
*ATF3*	NM_001674.3	Forward: 5′-GAGGATTTTGCTAACCTGAC-3′
		Reverse: 5′-TAGCTCTGCAATGTTCCTTC-3′
*CHOP*	NM_001195053.1	Forward: 5′-CATTGCCTTTCTCCTTCGGG-3′
		Reverse: 5′-GCCGTTCATTCTCTTCAGCT-3′
*Cyclin B1*	NM_031966.4	Forward: 5′-GTTGATACTGCCTCTCCAAG-3′
		Reverse: 5′-CTTAGTATAAGTGTTGTCAGTCAC-3′
*cyclin D1*	NM_053056	Forward: 5′-ATGGAACACCAGCTCCTGTGCTGC-3′
		Reverse: 5′-TCAGATGTCCACGTCCCGCACGTCGG-3′
*ATF4*	NM_001675.4	Forward: 5′-TTCCAGCAAAGCACCGCAAC-3′
		Reverse: 5′-AGGGCATCCAAGTCGAACTCCT-3′
*HIF-1a*	NM_001243084.1	Forward: 5′-GAACCTGATGCTTTAACT-3′
		Reverse: 5′-CAACTGATCGAAGGAACG-3′
*HO-1*	NM_002133.3	Forward: 5′-ATGCCCCAGGATTTGTCAGAG-3′
		Reverse: 5′-AGGGCTTTCTGGGCAATCTTT-3′
*LC3B*	NM_022818.5	Forward: 5′-AGCAGCATCCAACCAAAATC-3′
		Reverse: 5′-TGACAATTTCATCCCGAACG-3′
*Nrf-2*	NM_006164.5	Forward: 5′-CAGTCAGCGACGGAAAGAGT-3′
		Reverse: 5′-GGCTACCTGAGCAACAGAAG-3′
*p21*	NM_000389.4	Forward: 5′-CTGAGCCGCGACTGTGATGCG-3′
		Reverse: 5′-GGTCTGCCGCCGTTTTCGACC-3′
*p53*	NM_000546.5	Forward: 5′-CTCTGACTGTACCACCATCCACTA-3′
		Reverse: 5′-GAGTTCCAAGGCCTCATTCAGCTC-3′
*GAPDH*	NM_002046.7	Forward: 5′-CTTCATTGACCTCAACTAC-3′
		Reverse: 5′-GCCATCCACAGTCTTCTG-3′
*XBP-1*	NM_005080.4	Forward: 5′-CCTTGTAGTTGAGAACCAGG-3′
		Reverse: 5′-GGGGCTTGGTATATATGTGG-3′
*eIF2a*	NM_004094.5	Forward: 5′-ACCTCAGAATGCCGGGTCTA-3′
		Reverse: 5′-GTGGGGTCAAGCGCCTATTA-3′
*KEAP1*	NM_203500.2	Forward: 5′-CCCCAACCGACAACCAAGAC-3′
		Reverse: 5′-CCCTCAATGGACACCACCTC-3′
*p62*	NM_003900.5	Forward: 5′-CCGTGAAGGCCTACCTTCTG-3′
		Reverse: 5′-GCACTTGTAGCGGGTTCCTA-3′

### Statistical analysis

The values are expressed as the means ± SDs of at least three independent experiments. All the comparisons between groups were conducted using Student’s *t*-tests, and the comparison between multiple groups was conducted using analysis of variance (ANOVA) with SPSS 20.0 for Windows (SPSS, Chicago, IL). The statistical significance was set to *p < *0.05.

## Supplementary Material

RB original data.pdf

## Data Availability

The data generated during the current study are available from the corresponding author on reasonable request.
