# Supplementary material for: Remimazolam induced cytotoxicity mediated through multiple stress pathways and acted synergistically with tyrosine kinase inhibitors in hepatocellular carcinoma
Source: Redox Rep. 2025 Mar 7;30(1):2475696. doi: 10.1080/13510002.2025.2475696 (PMC11892054; doi:10.1080/13510002.2025.2475696)
Supplement: RB original data.pdf [file YRER_A_2475696_SM2043.pdf]

Figure 2C:

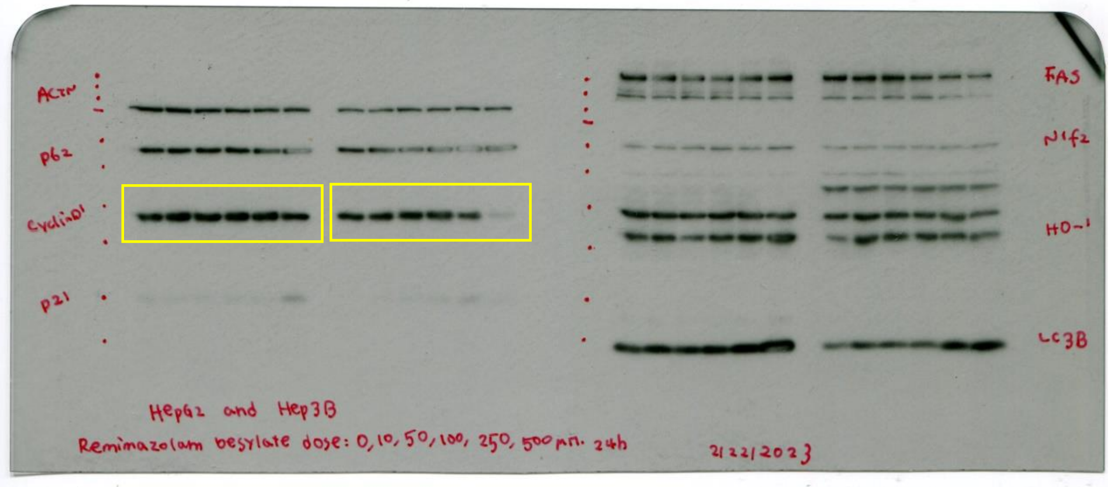
ACTN
p62
CyclinD1
p21
FAS
Nrf2
HO-1
LC3B
HepG2 and Hep3B
Remimazolam besylate dose: 0, 10, 50, 100, 250, 500 μM. 24h
2/22/2023

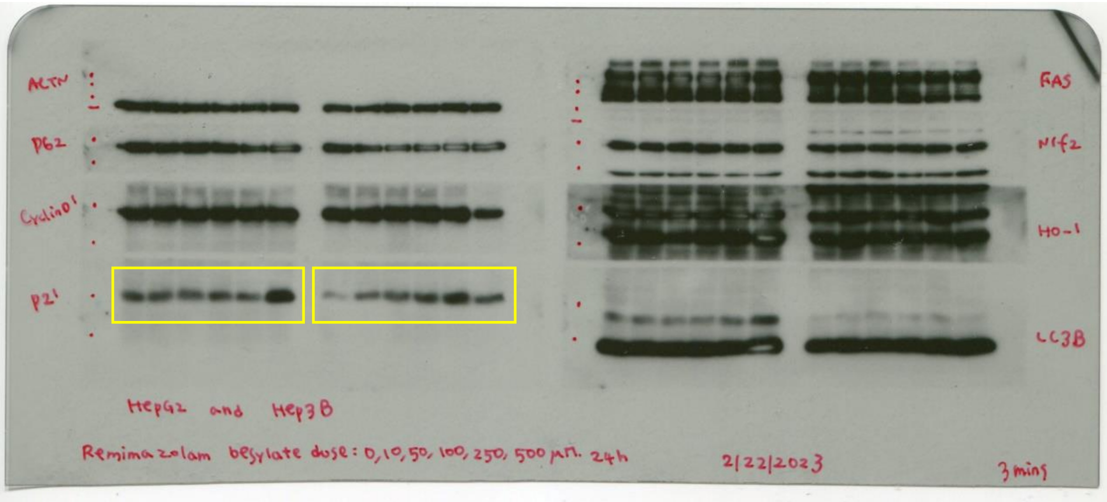
ACTN
P62
CyclinD1
P21
FAS
Nrf2
HO-1
LC3B
HepG2 and Hep3B
Remimazolam besylate dose: 0, 10, 50, 100, 250, 500 μM. 24h
2/22/2023
3 mins

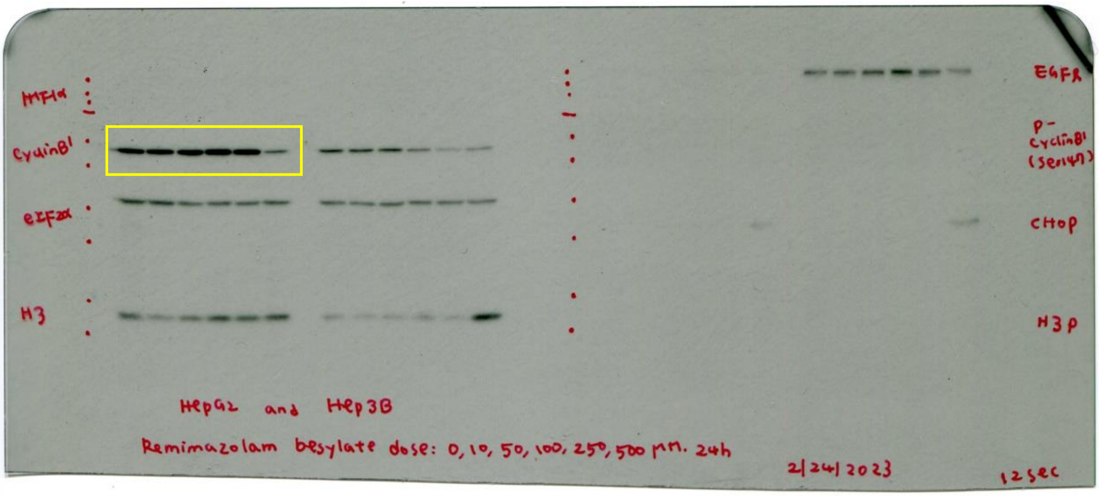
HIF1α
CyclinB1
eIF2α
H3
EGFR
P-CyclinB1 (Ser147)
CHOP
H3p
HepG2 and Hep3B
Remimazolam besylate dose: 0, 10, 50, 100, 250, 500 μM. 24h
2/24/2023
12sec

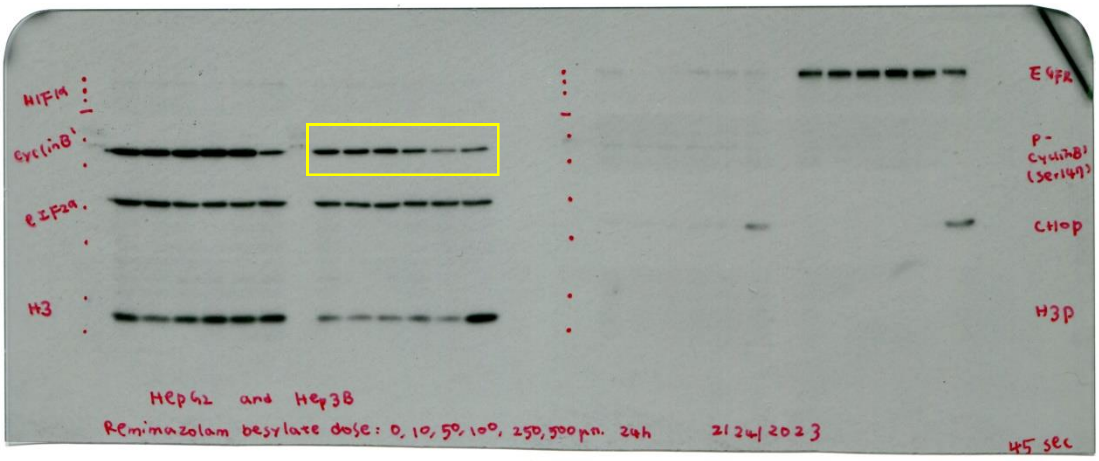
cyclinB1
eIF2α
H3
EGFR
CHOP
H3p
HepG2 and Hep3B
Remimazolam besylate dose: 0, 10, 50, 100, 250, 500 μM. 24h
2/24/2023
45 sec

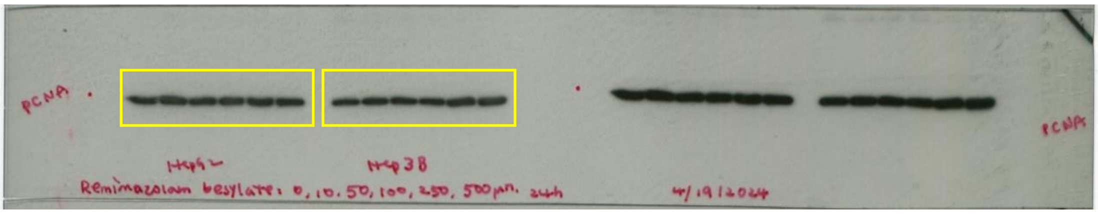
PCNA
HepG2
Hep3B
Remimazolam besylate: 0, 10, 50, 100, 250, 500 μM. 24h
4/19/2024

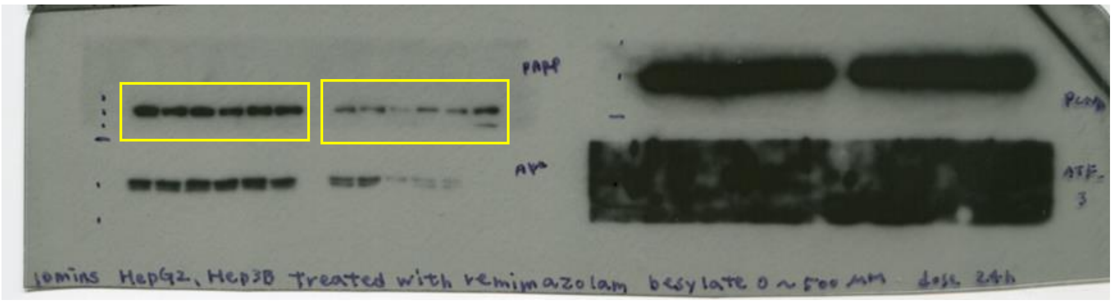
PARP
PCNA
ATF-3
10mins HepG2, Hep3B treated with remimazolam besylate 0 ~ 500 μM dose 24h

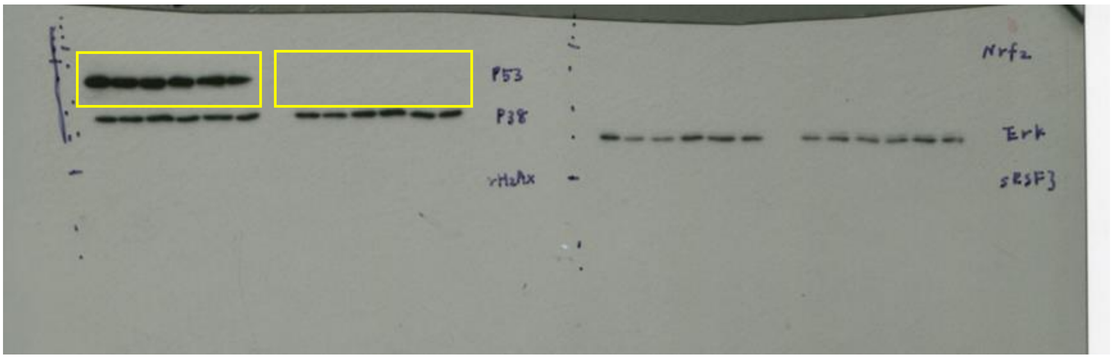
P53
P38
γH2AX
Nrf2
Erk
SRSF3

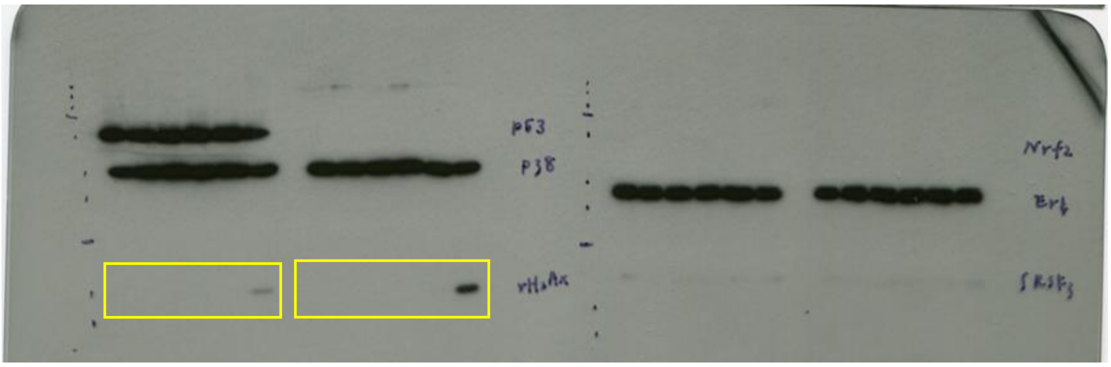
P38
Nrf2

Figure 2D:

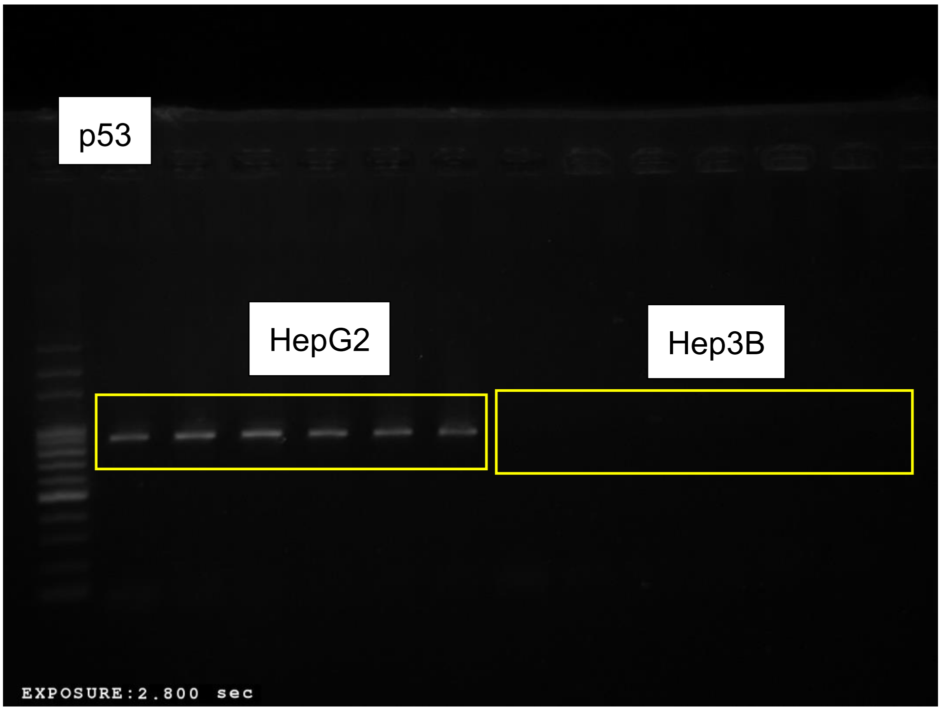
p53
HepG2
Hep3B
EXPOSURE:2.800 sec

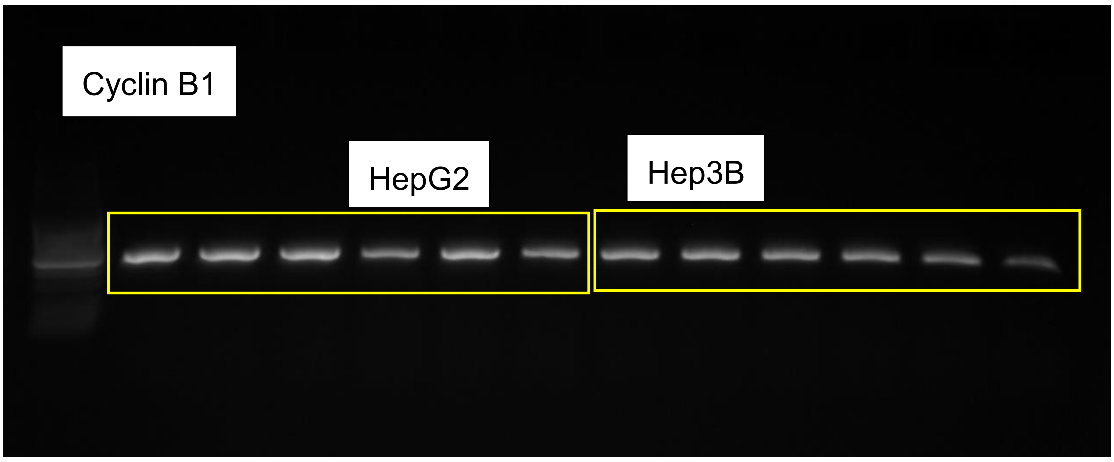
Cyclin B1
HepG2
Hep3B

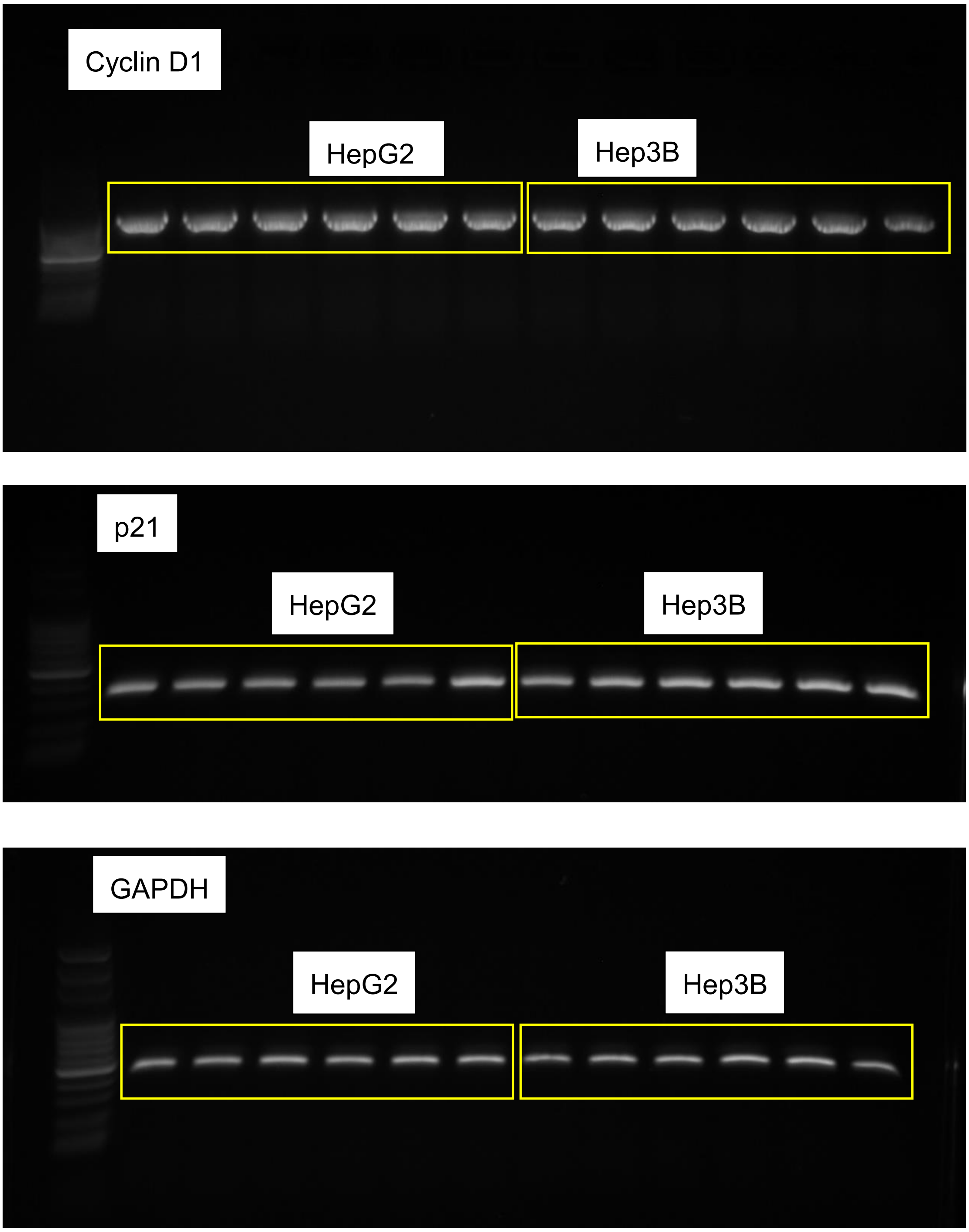

Figure 4:

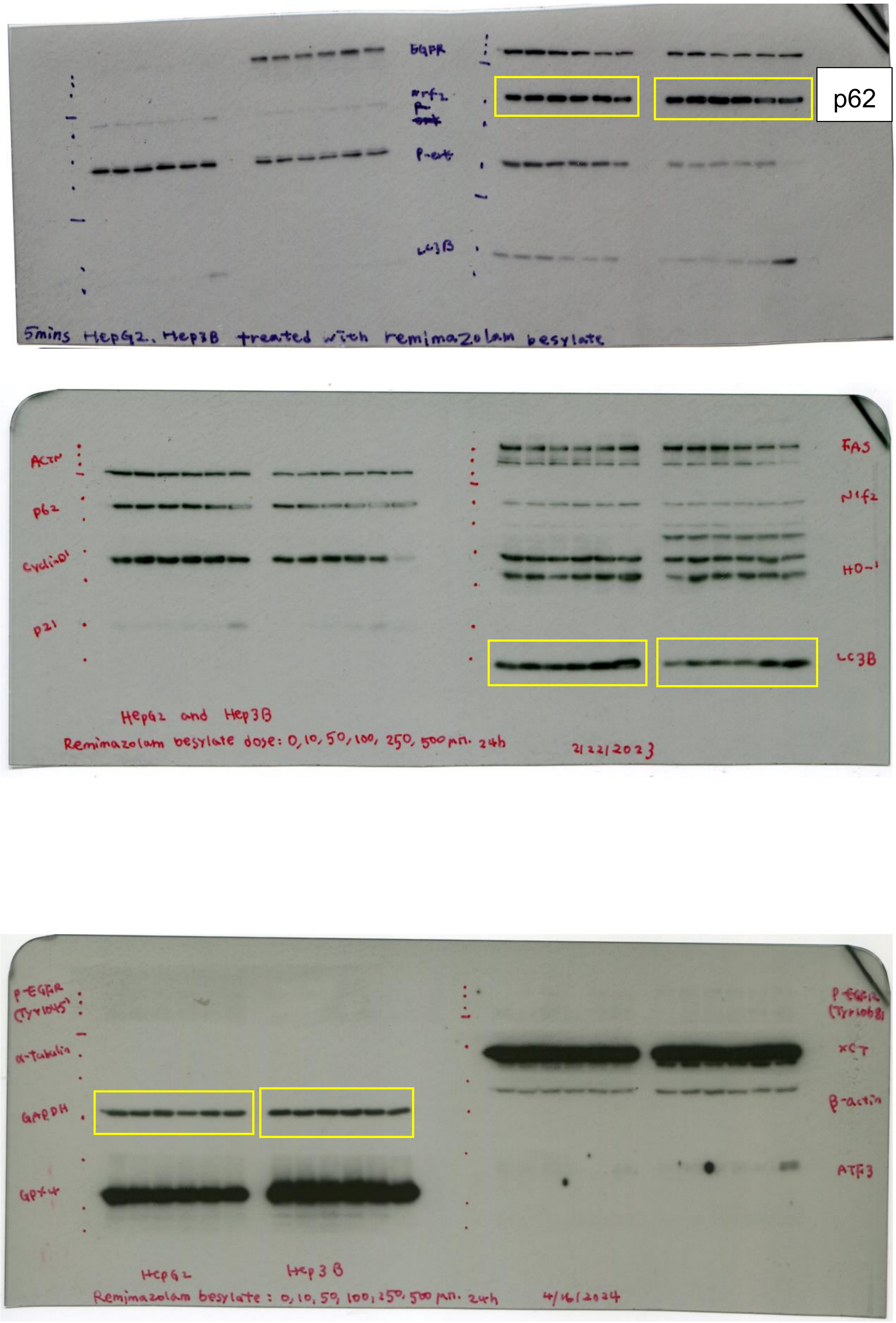
p62
EGFR
Nrf2
LC3B
5mins HepG2, Hep3B treated with remimazolam besylate
Actin
p62
Cyclin D1
p21
FAS
Nrf2
HO-1
LC3B
HepG2 and Hep3B
Remimazolam besylate dose: 0, 10, 50, 100, 250, 500 μM. 24h
2/22/2023
p-EGFR (Tyr1045)
α-Tubulin
GAPDH
GPX4
p-EGFR (Tyr1068)
xCT
β-actin
ATF3
HepG2
Hep3B
Remimazolam besylate: 0, 10, 50, 100, 250, 500 μM. 24h
4/16/2024

Figure 5:

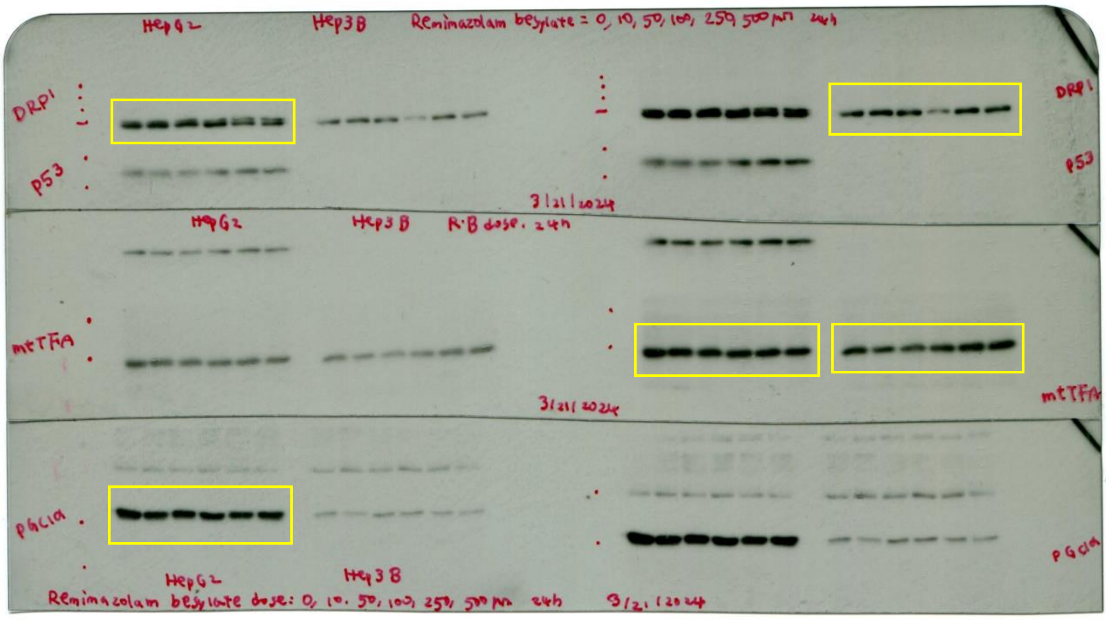
HepG2
Hep3B
Remimazolam besylate = 0, 10, 50, 100, 250, 500 μM 24h
DRP1
p53
3/21/2024
HepG2
Hep3B
R·B dose. 24h
mtTFA
3/21/2024
PGC1α
HepG2
Hep3B
Remimazolam besylate dose: 0, 10, 50, 100, 250, 500 μM 24h
3/21/2024

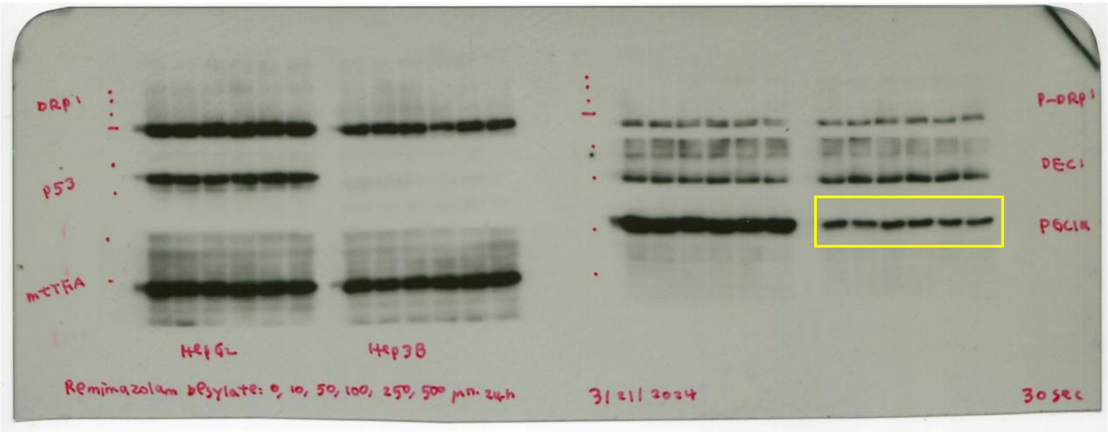
DRP1
p53
mtTFA
HepG2
Hep3B
Remimazolam besylate: 0, 10, 50, 100, 250, 500 μM. 24h
3/21/2024
P-DRP1
DEC1
PGC1α
30 sec

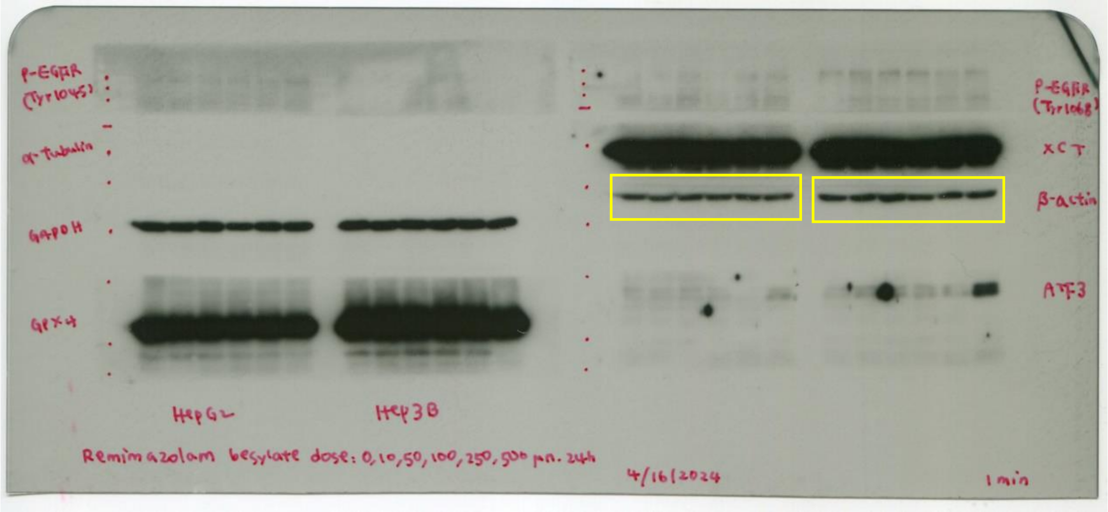
P-EGFR (Tyr1045)
α-Tubulin
GAPDH
GPX4
HepG2
Hep3B
Remimazolam besylate dose: 0, 10, 50, 100, 250, 500 μM. 24h
4/16/2024
P-EGFR (Tyr1068)
xCT
β-actin
ATF3
1 min

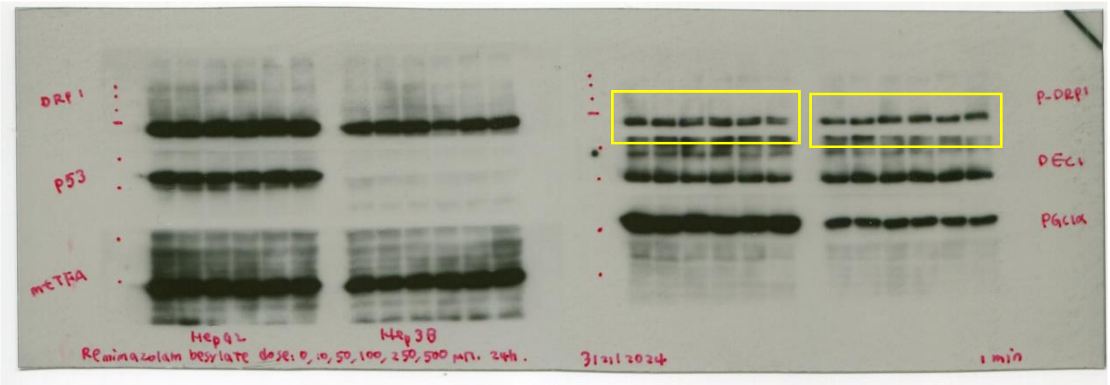
DRP1
p53
mtTFA
HepG2
Hep3B
3/21/2024
1 min
P-DRP1
PGC1α

Figure 6:

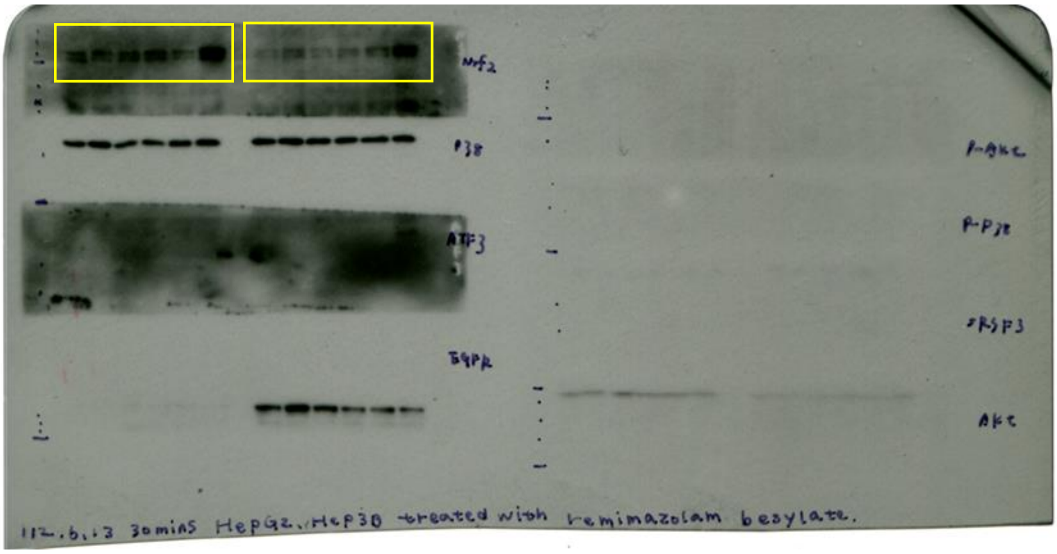
Nrf2
P38
ATF3
P-Akt
P-P38
Akt

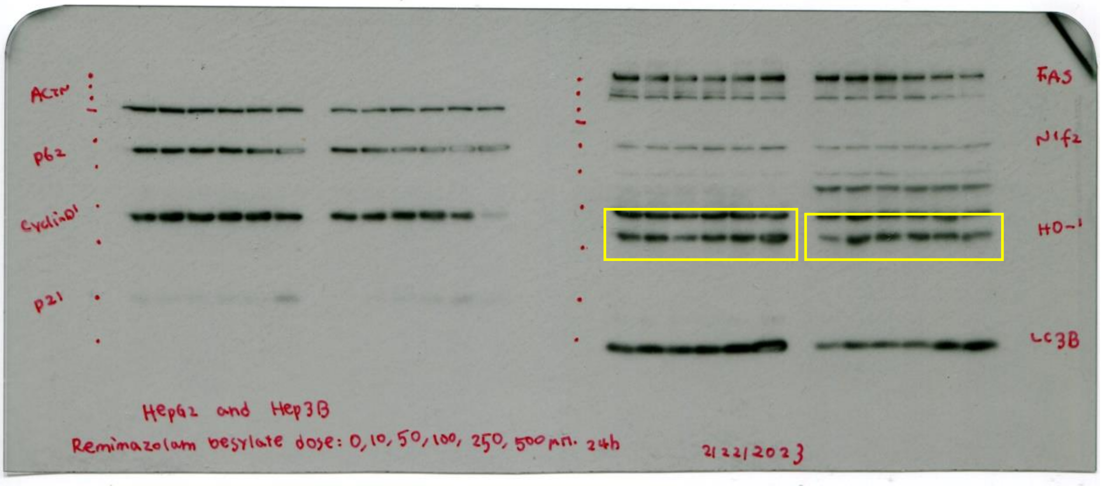
Actin
p62
CyclinD1
p21
HepG2 and Hep3B
2/22/2023
FAS
Nrf2
HO-1
LC3B

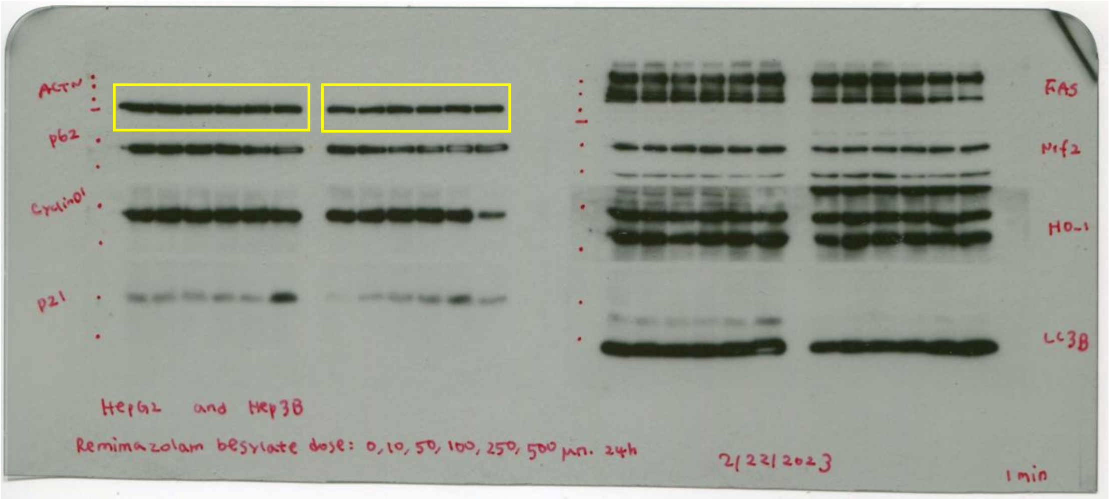
ACTN
p62
CyclinD1
p21
FAS
Nrf2
HO-1
LC3B
HepG2 and Hep3B
Remimazolam besylate dose: 0, 10, 50, 100, 250, 500 μM. 24h
2/22/2023
1 min

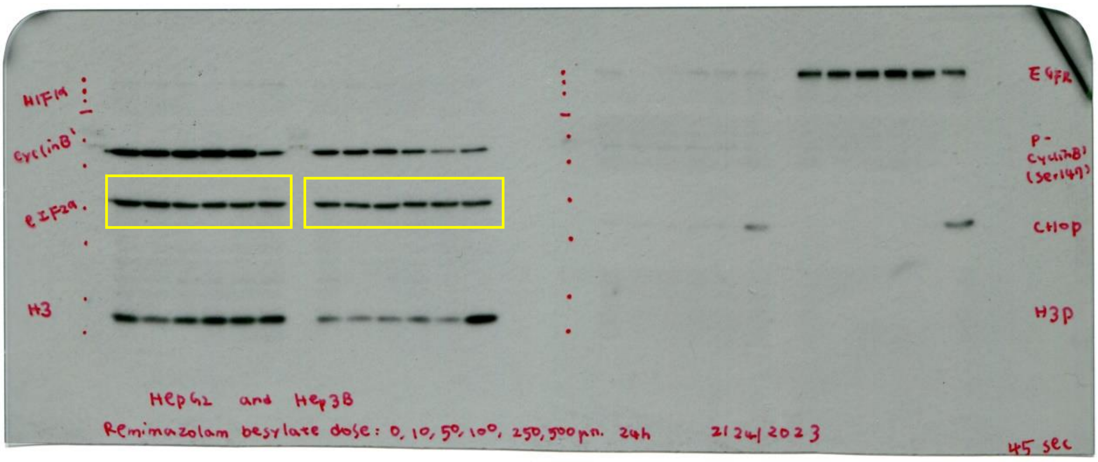
HIF1α
CyclinB1
E2F1α
H3
EGFR
P-CyclinB1 (ser147)
CHOP
H3P
HepG2 and Hep3B
Remimazolam besylate dose: 0, 10, 50, 100, 250, 500 μM. 24h
2/24/2023
45 sec

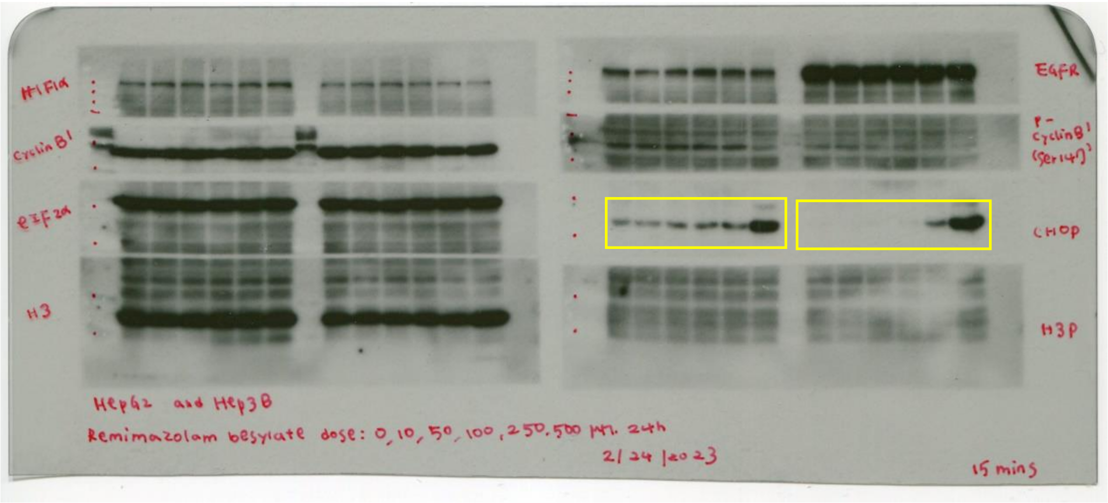
HIF1α
CyclinB1
E2F1α
H3
EGFR
P-CyclinB1 (ser147)
CHOP
H3P
HepG2 and Hep3B
Remimazolam besylate dose: 0, 10, 50, 100, 250, 500 μM. 24h
2/24/2023
15 mins

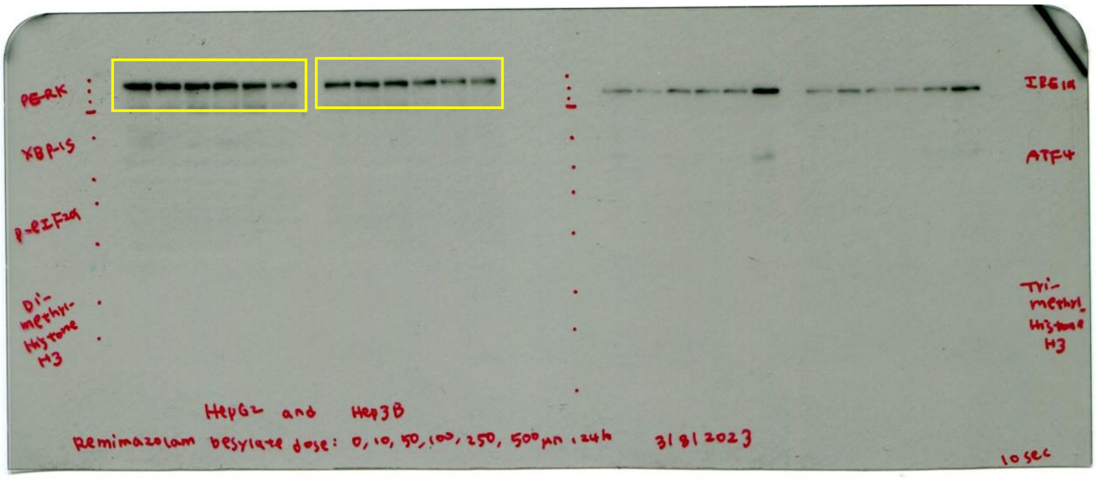
PERK
XBP-1S
p-eIF2α
Di-methyl-Histone H3
IRE1α
ATF4
Tri-methyl-Histone H3
HepG2 and Hep3B
Remimazolam besylate dose: 0, 10, 50, 100, 250, 500μM, 24h
3/8/2023
10sec

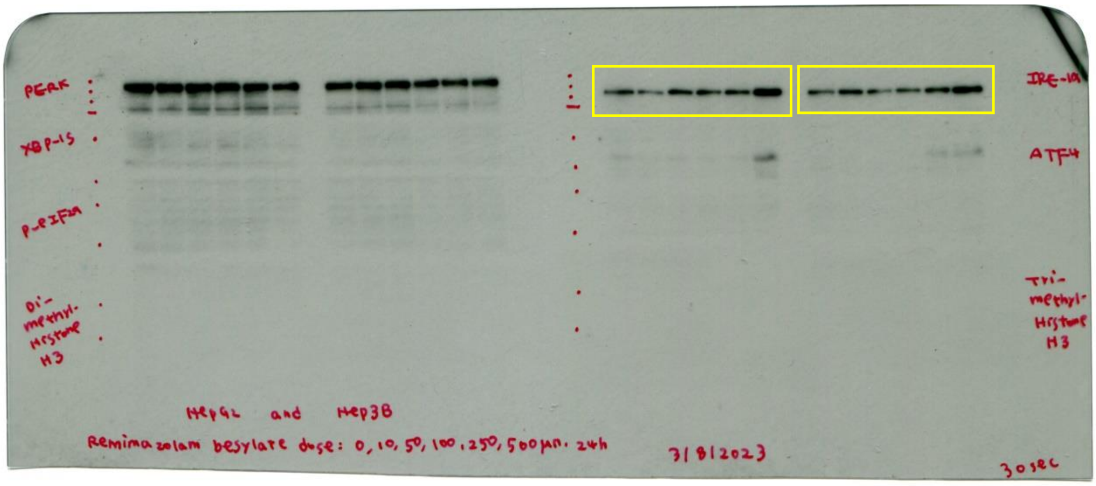
PERK
XBP-1S
p-eIF2α
Di-methyl-Histone H3
IRE-1α
ATF4
Tri-methyl-Histone H3
HepG2 and Hep3B
Remimazolam besylate dose: 0, 10, 50, 100, 250, 500μM, 24h
3/8/2023
30sec

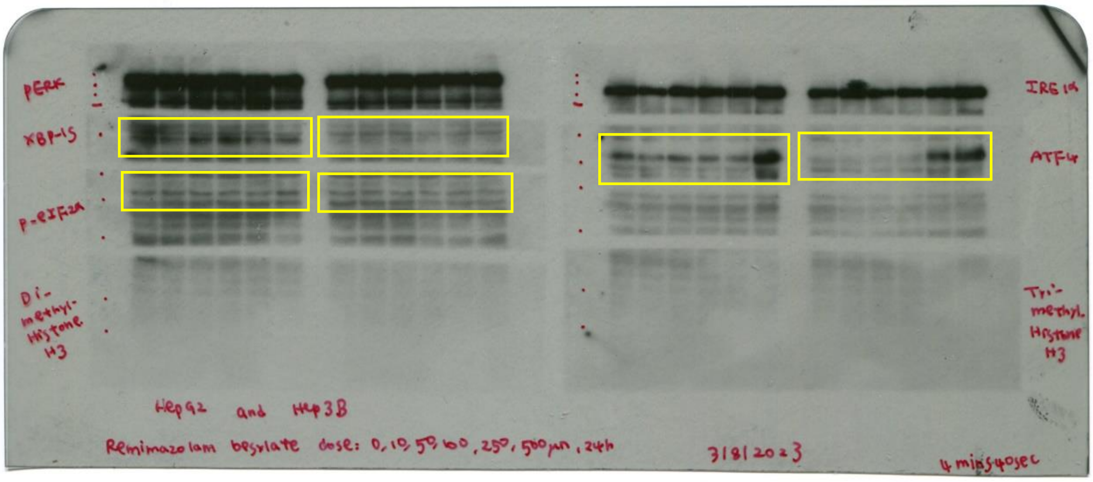
PERK
XBP-1S
p-eIF2α
Di-methyl-Histone H3
IRE1α
ATF4
Tri-methyl-Histone H3
HepG2 and Hep3B
Remimazolam besylate dose: 0, 10, 50, 100, 250, 500μM, 24h
3/8/2023
4mins40sec

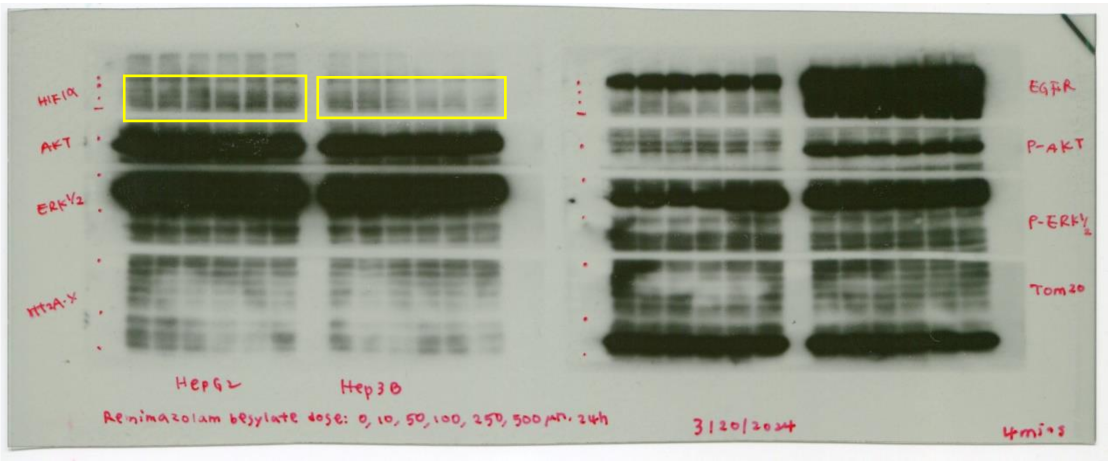
HIF1α
AKT
ERK1/2
EGFR
P-AKT
P-ERK1/2
Tom20
HepG2
Hep3B
Remimazolam besylate dose: 0, 10, 50, 100, 250, 500 μM, 24h
3/20/2024
4mins

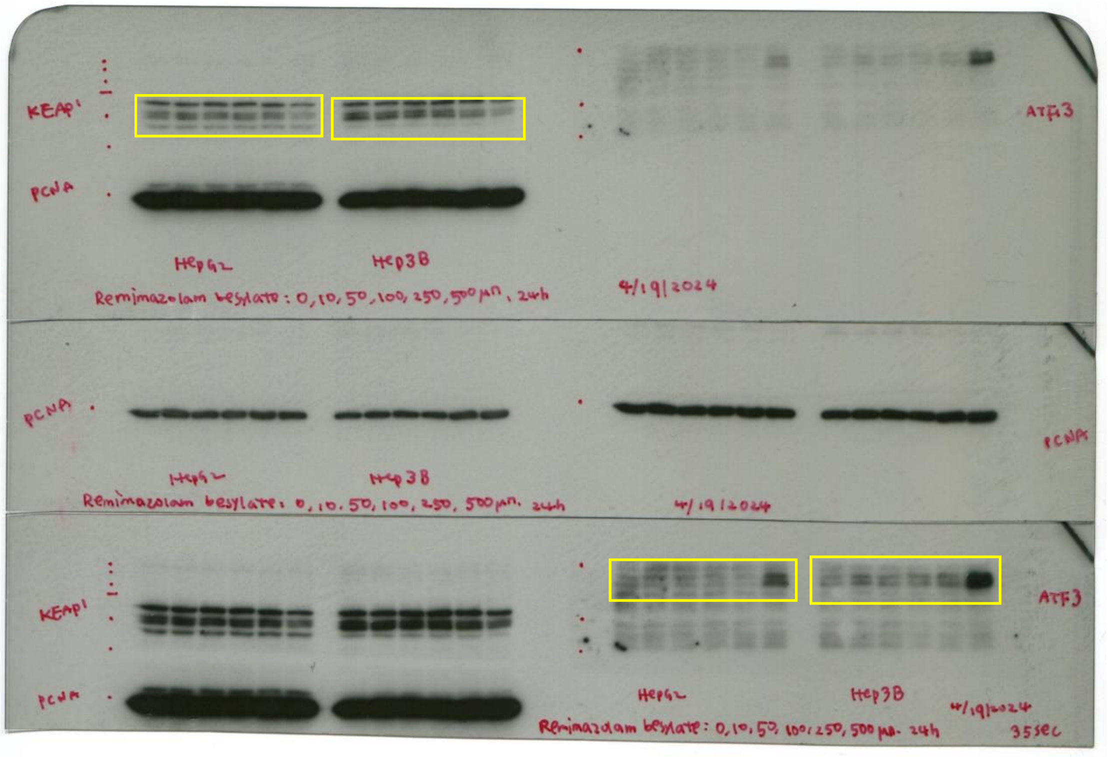
KEAP1
PCNA
ATF3
HepG2
Hep3B
Remimazolam besylate: 0, 10, 50, 100, 250, 500 μM, 24h
4/19/2024
PCNA
35sec

Figure 6C:

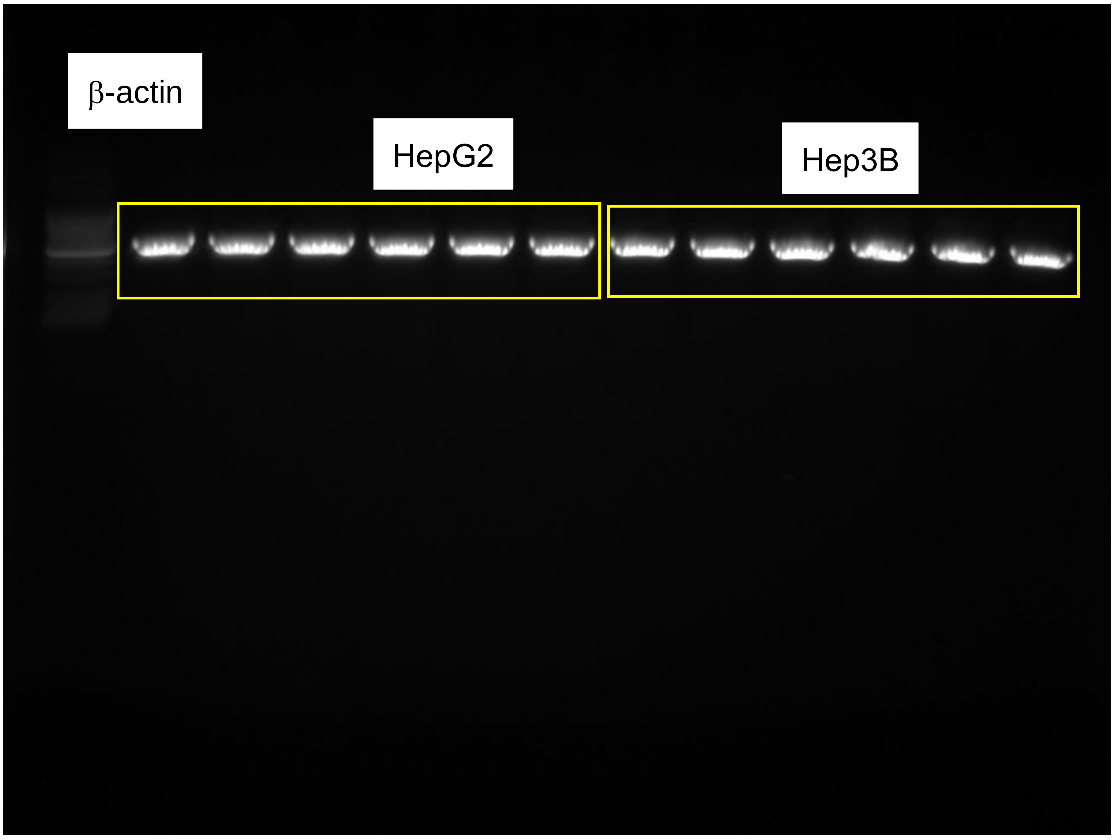
β-actin
HepG2
Hep3B

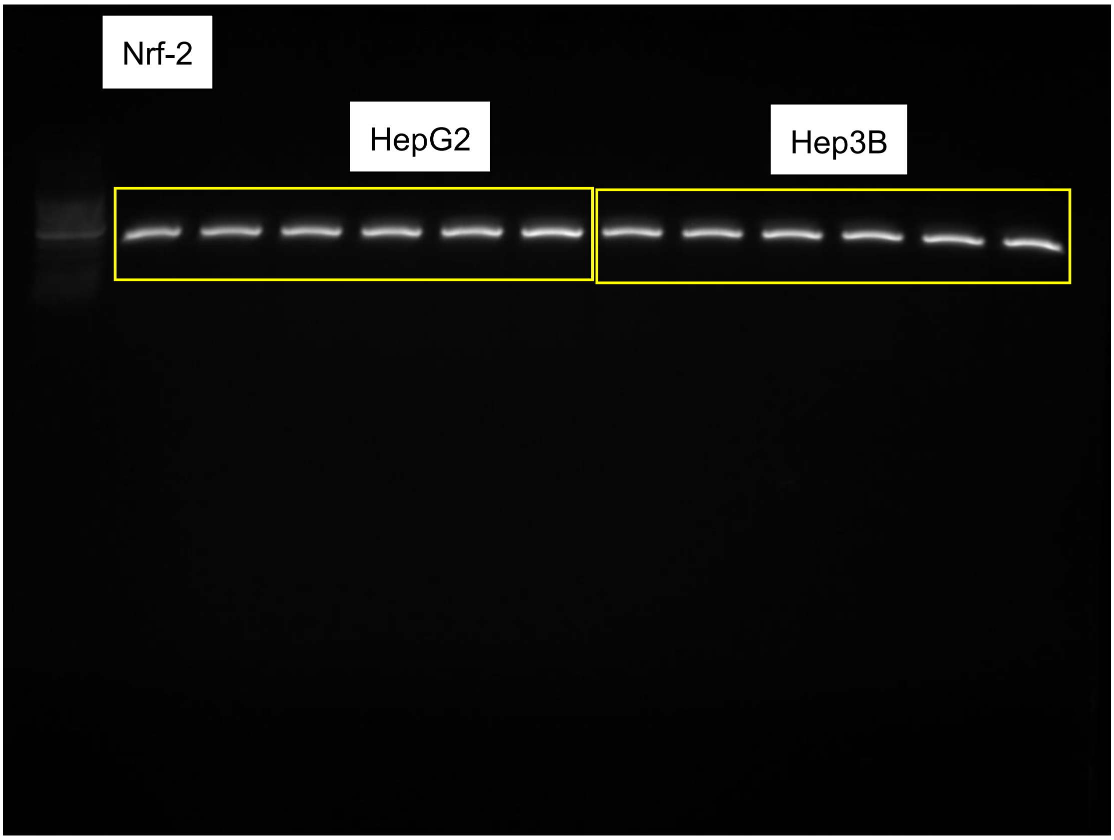
Nrf-2
HepG2
Hep3B

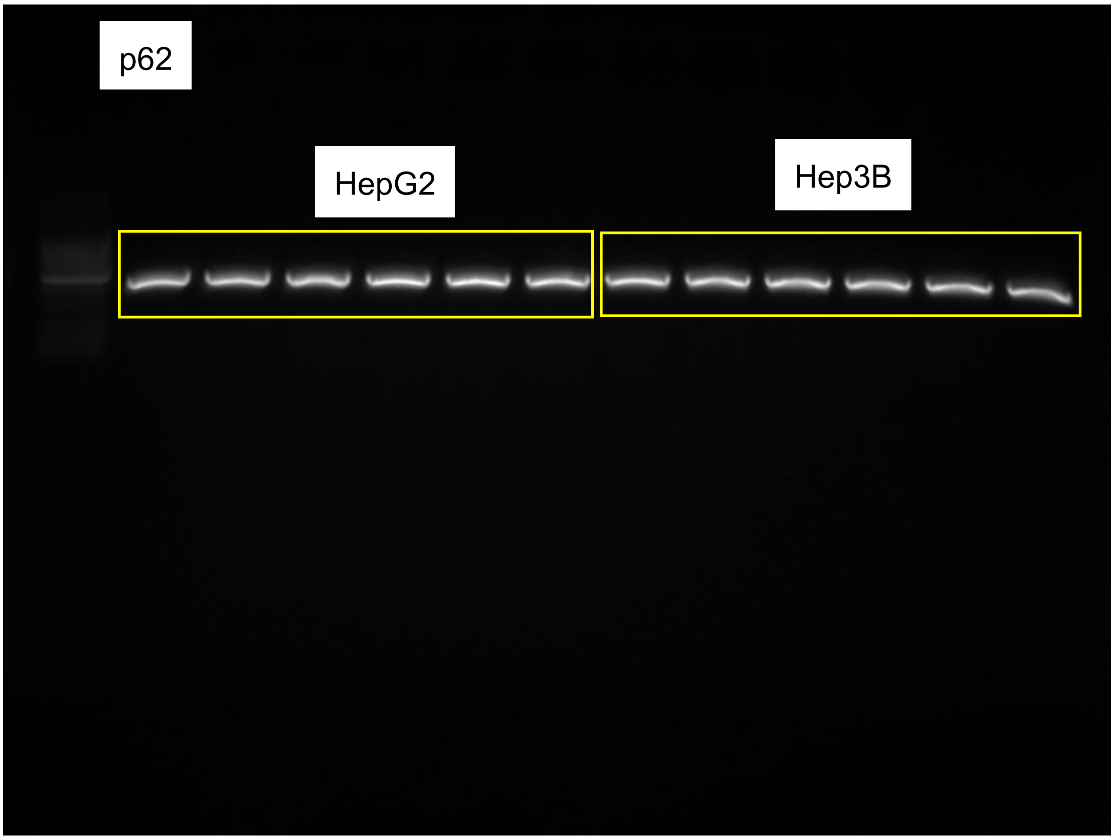
p62
HepG2
Hep3B

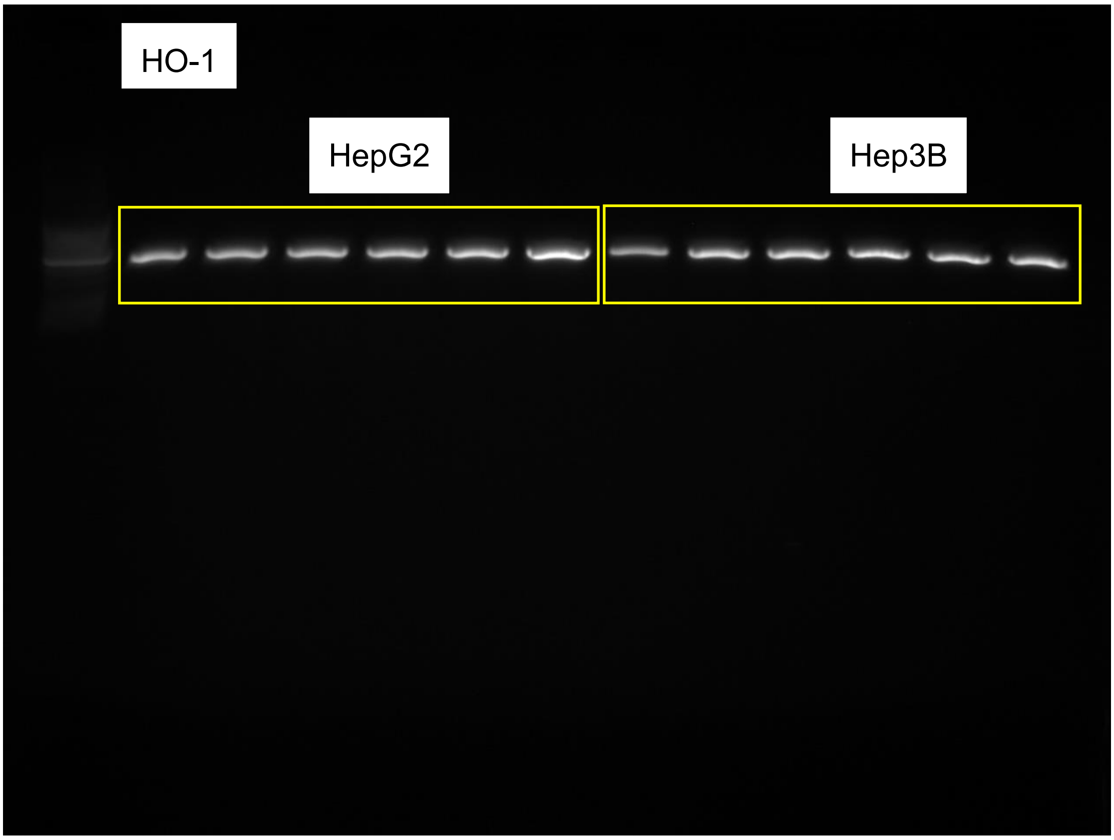
HO-1
HepG2
Hep3B

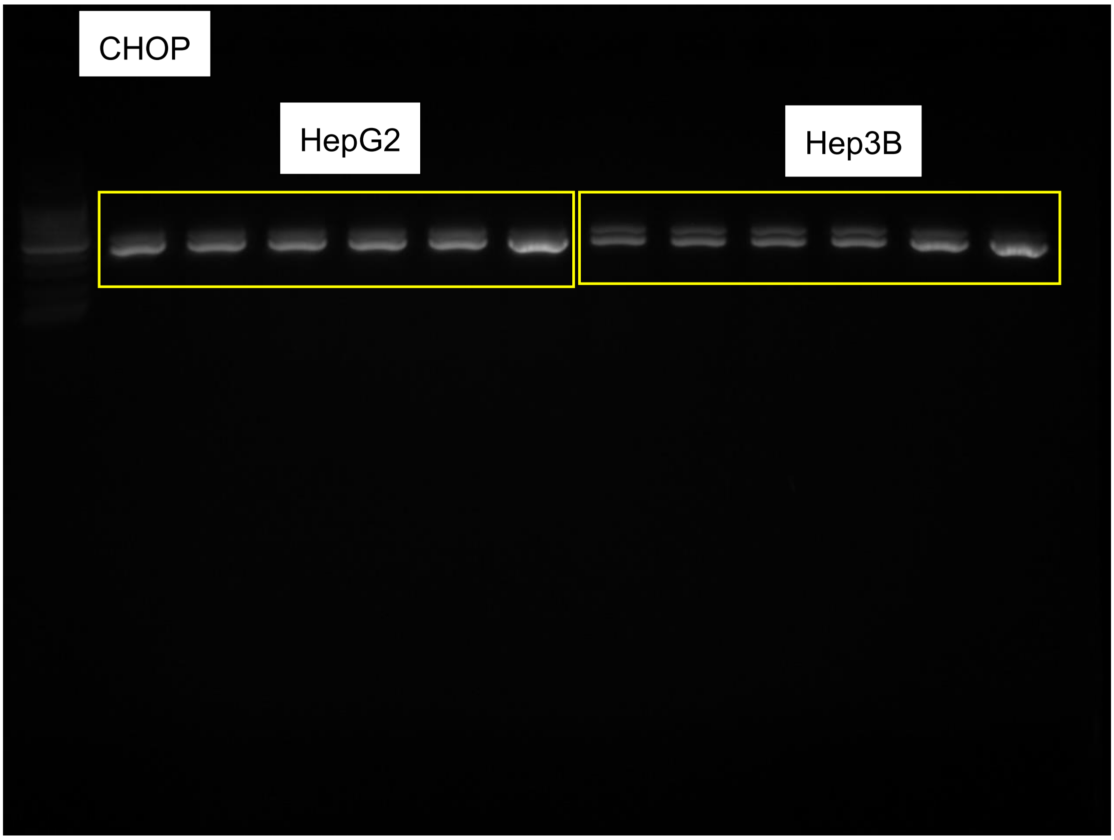
CHOP
HepG2
Hep3B

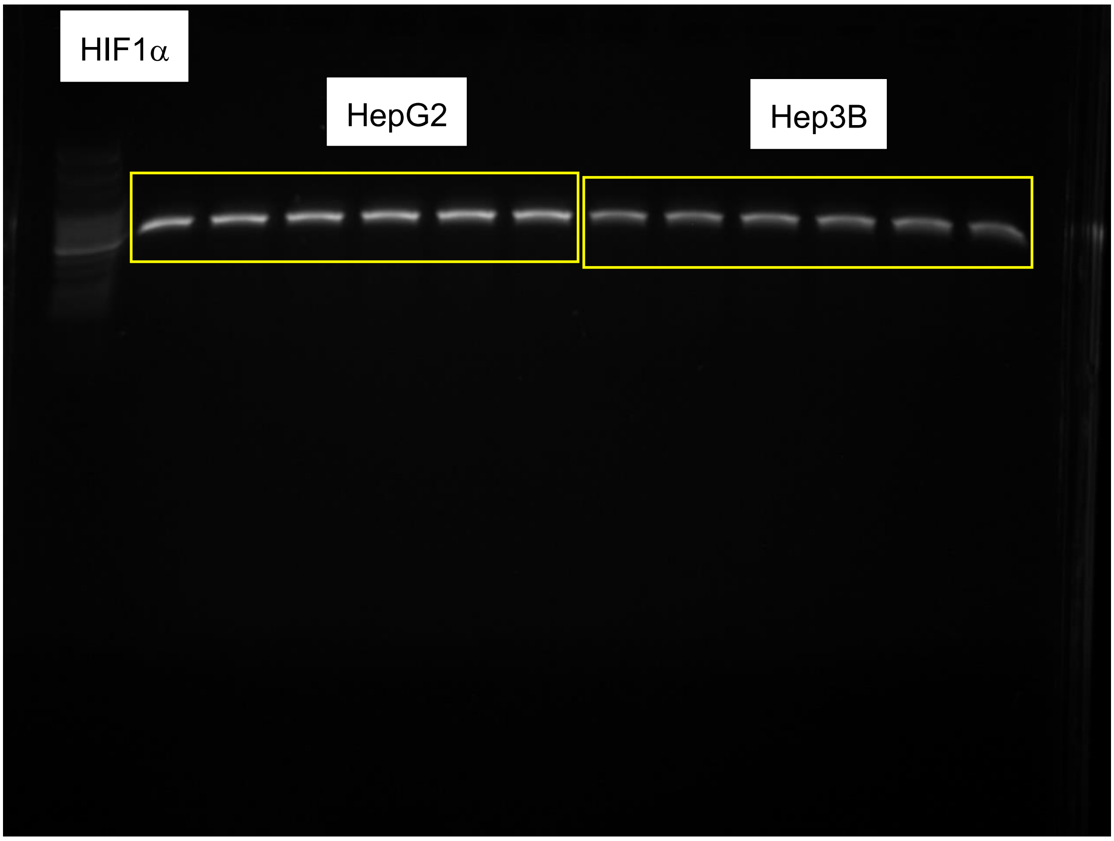
HIF1α
HepG2
Hep3B

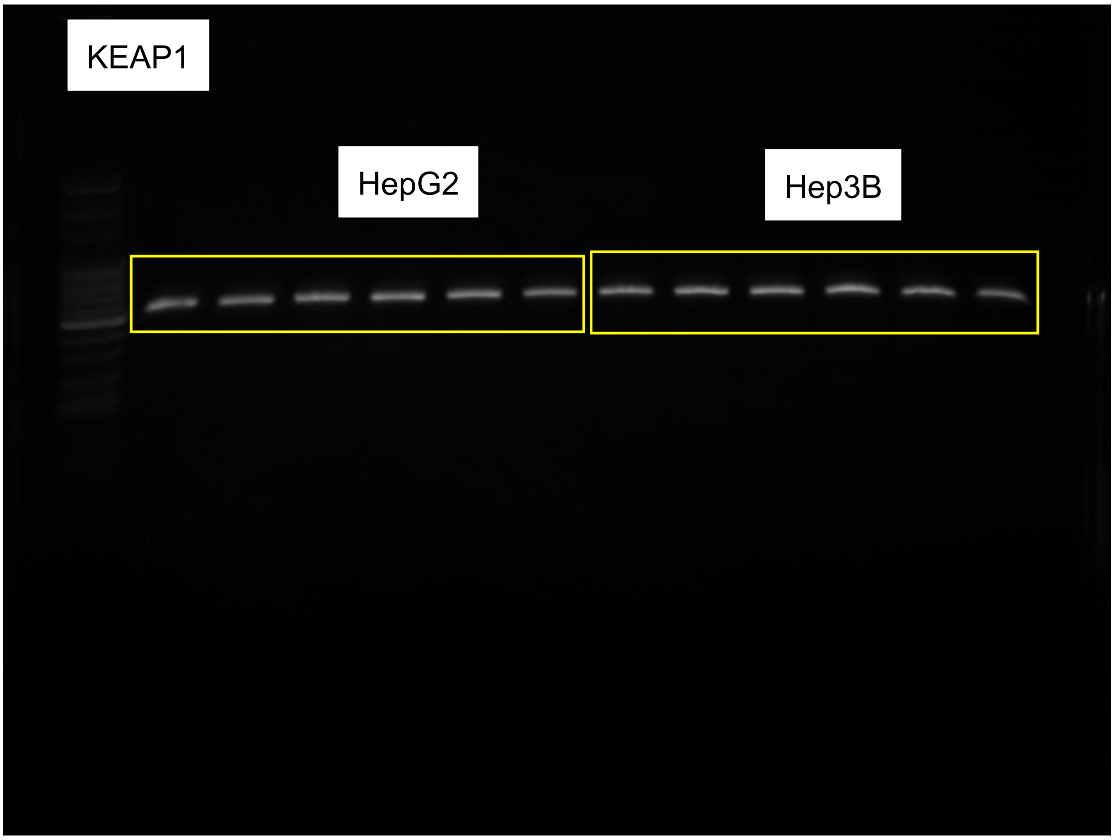
KEAP1
HepG2
Hep3B

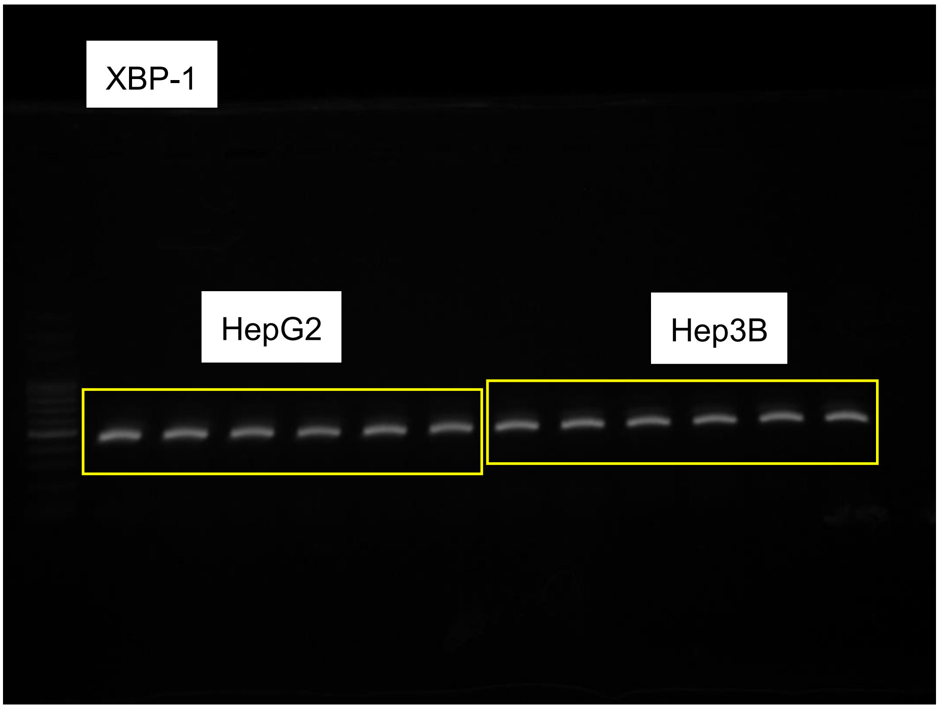
XBP-1
HepG2
Hep3B

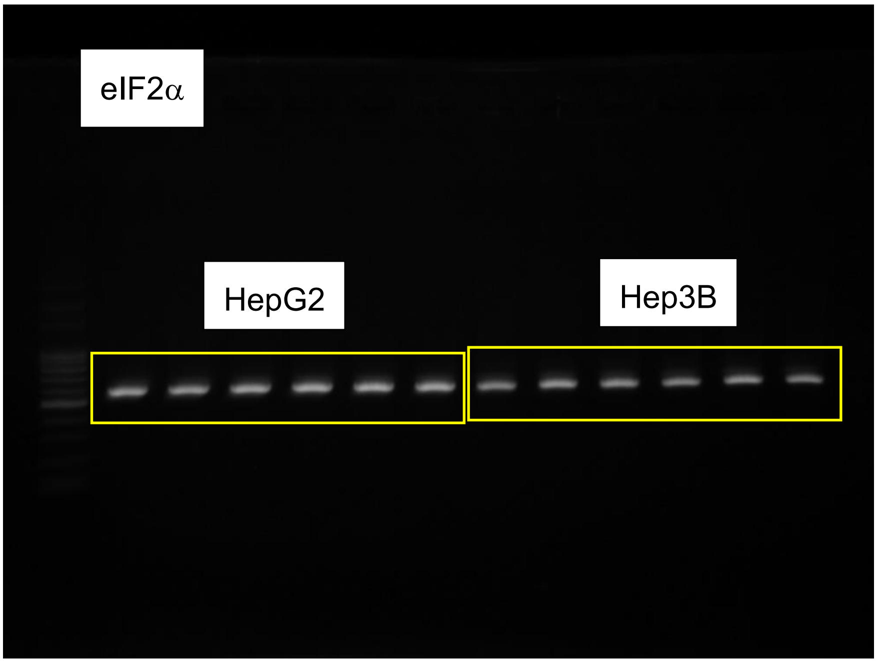
eIF2α
HepG2
Hep3B

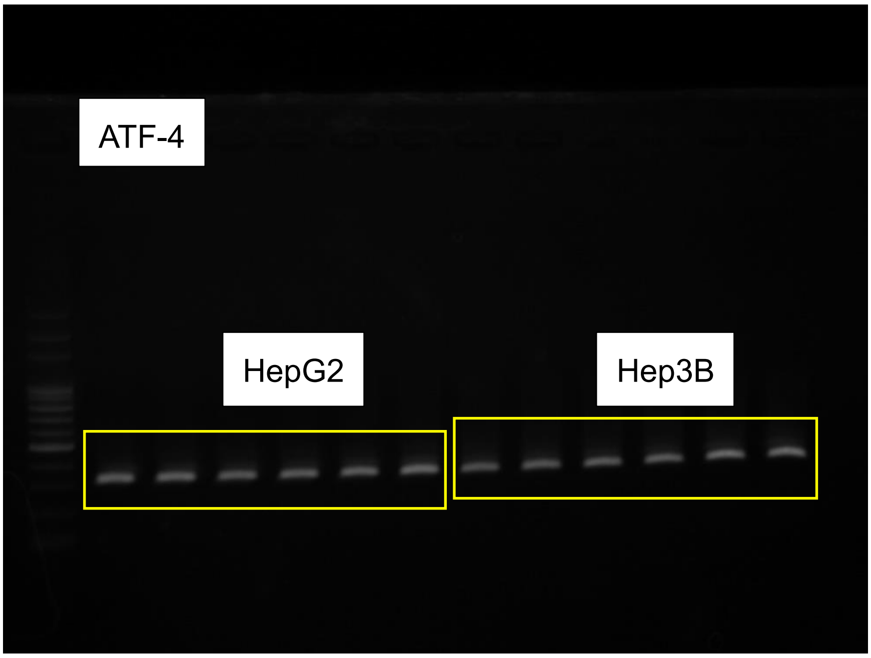
ATF-4
HepG2
Hep3B

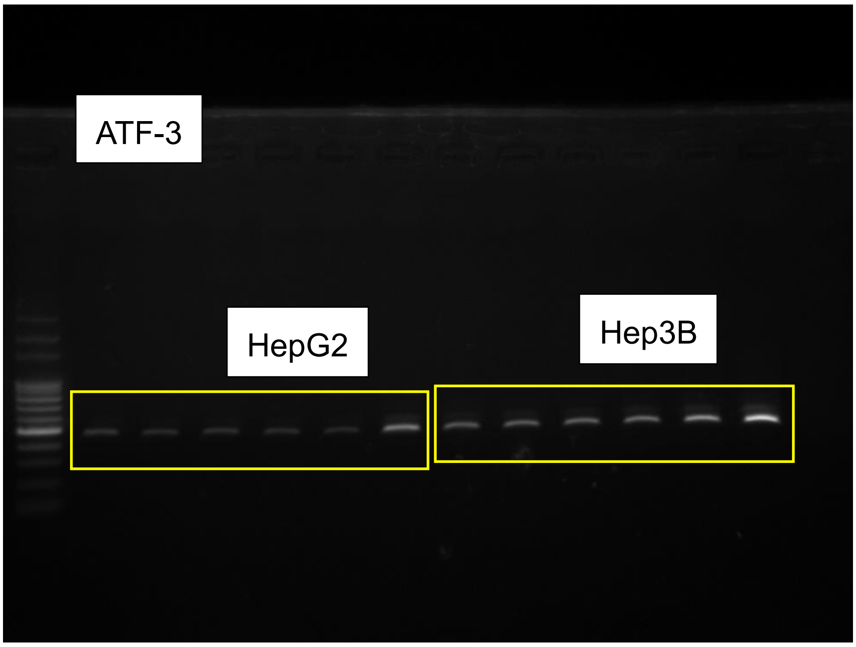
ATF-3
HepG2
Hep3B

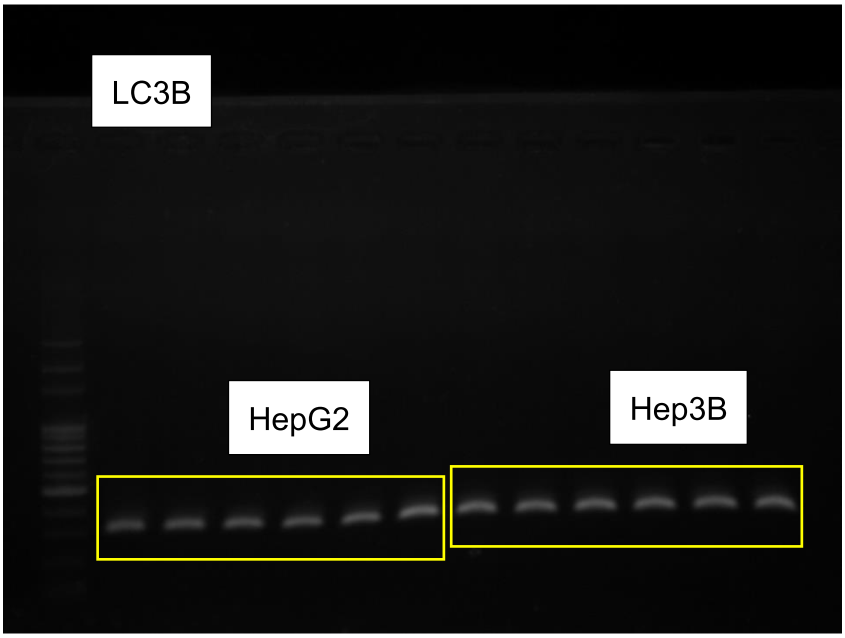
LC3B
HepG2
Hep3B

Figure 7A:

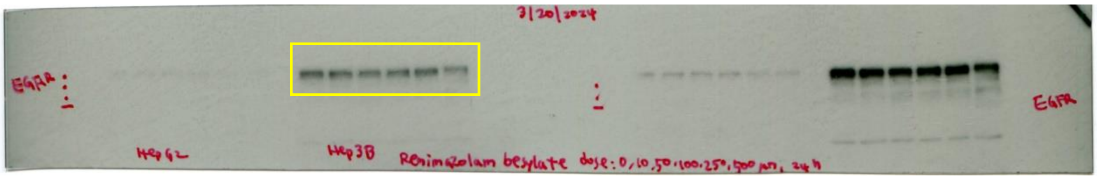
3/20/2024
EGFR
HepG2
Hep3B
Remimazolam besylate dose: 0, 10, 50, 100, 250, 500 μM, 24h
EGFR

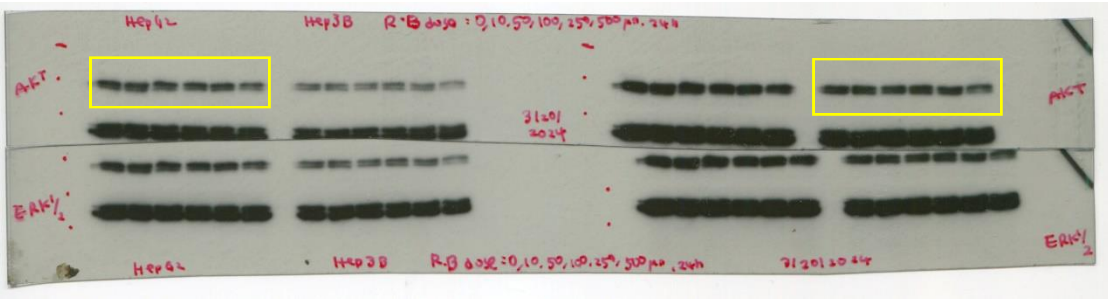
HepG2
Hep3B
R-B dose : 0, 10, 50, 100, 250, 500 μM, 24h
AKT
3/20/2024
AKT
ERK1/2
ERK1/2
HepG2
Hep3B
R-B dose : 0, 10, 50, 100, 250, 500 μM, 24h
3/20/2024

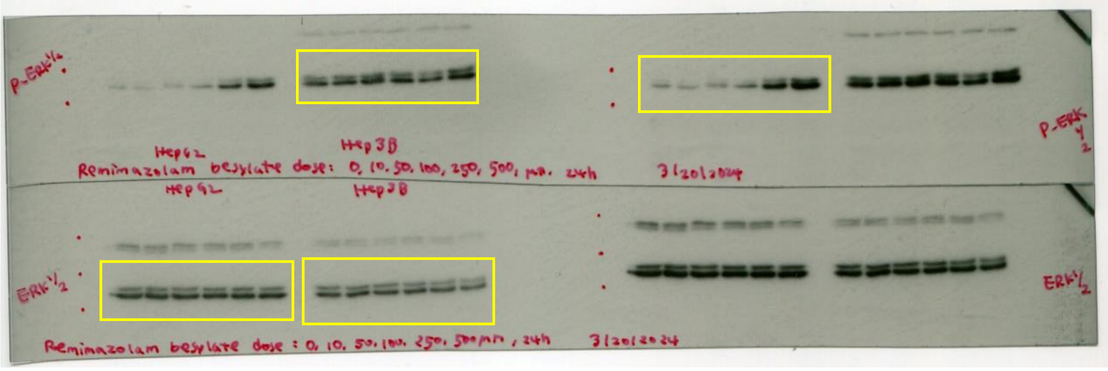
P-ERK1/2
P-ERK1/2
HepG2
Hep3B
Remimazolam besylate dose: 0, 10, 50, 100, 250, 500 μM, 24h
3/20/2024
HepG2
Hep3B
ERK1/2
ERK1/2
Remimazolam besylate dose : 0, 10, 50, 100, 250, 500 μM, 24h
3/20/2024

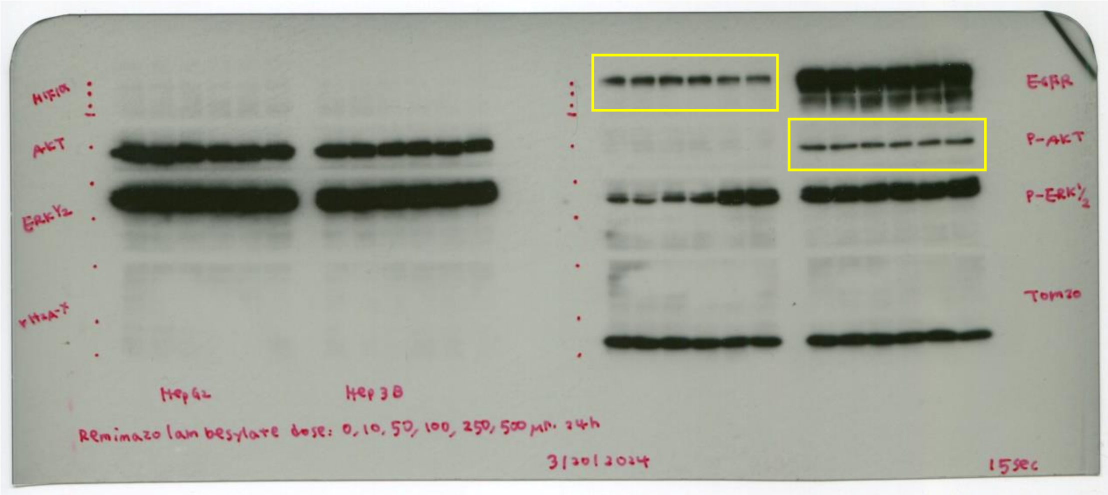
HIF1α
AKT
ERK1/2
γH2A.X
EGFR
P-AKT
P-ERK1/2
Tom20
HepG2
Hep3B
Remimazolam besylate dose: 0, 10, 50, 100, 250, 500 μM, 24h
3/20/2024
15sec

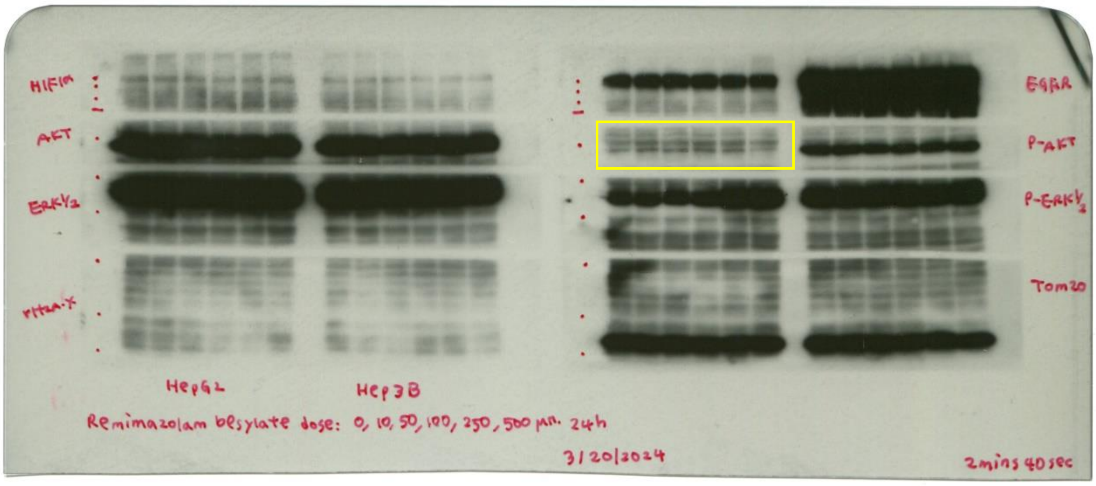
HIF1α
AKT
ERK1/2
γH2A.X
EGFR
P-AKT
P-ERK1/2
Tom20
HepG2
Hep3B
Remimazolam besylate dose: 0, 10, 50, 100, 250, 500 μM, 24h
3/20/2024
2mins 40sec

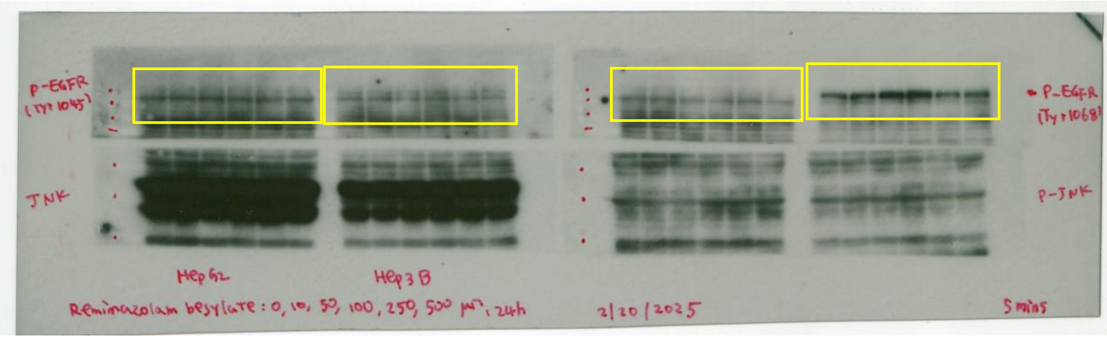
P-EGFR (Tyr 1045)
JNK
P-EGFR (Tyr1068)
P-JNK
HepG2
Hep3B
Remimazolam besylate: 0, 10, 50, 100, 250, 500 μM, 24h
2/20/2025
5 mins

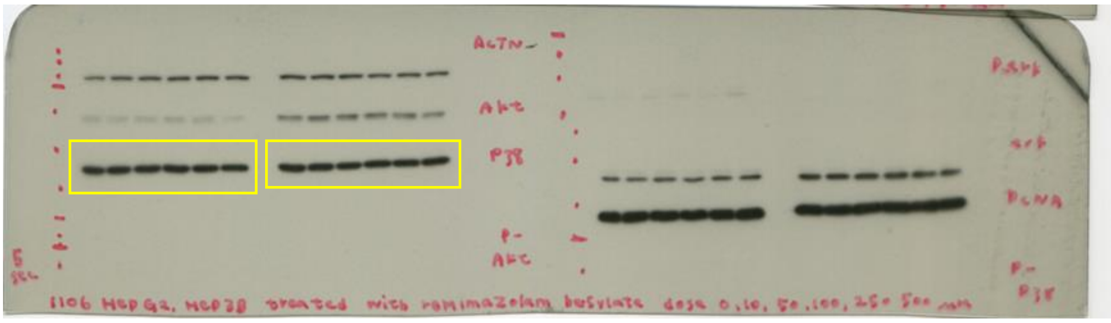
ACTN
Akt
P38
P-Akt
P-erk
erk
PCNA
P-P38
5 sec

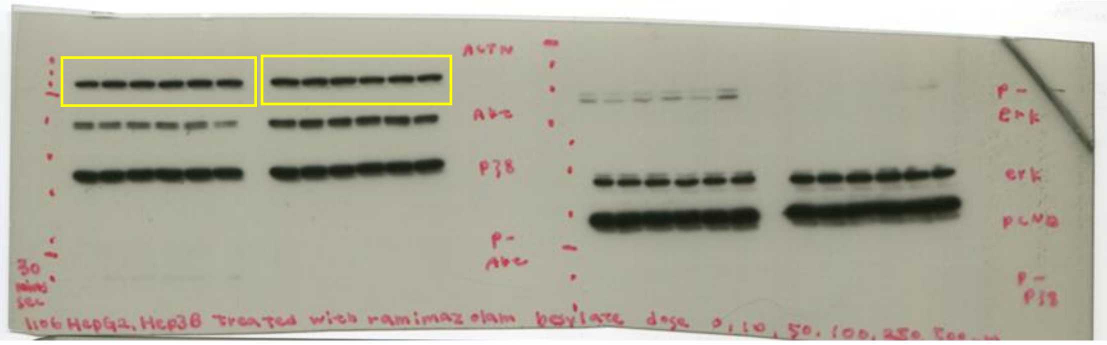
ACTN
Akt
P38
P-Akt
P-erk
erk
PCNA
P-P38

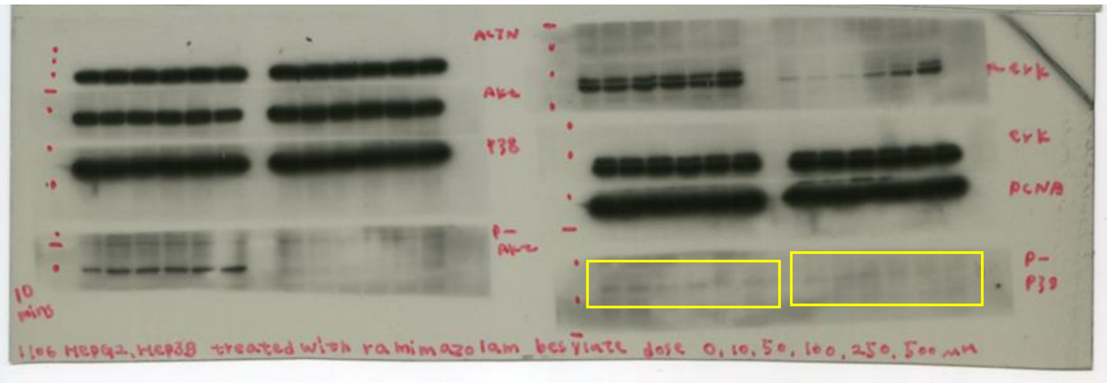
ACTN
Akt
P38
P-Akt
P-erk
erk
PCNA
P-P38
10 mins

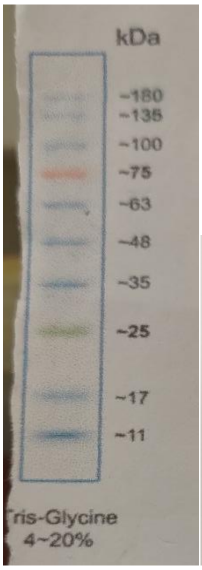

BIOMAN SCIENTIFIC CO,LTD

BIOMAN SCIENTIFIC CO.,LTD

Prestained Protein Ladder

Cat No: PREP1025G **Size:** 0.25ml / 0.5 ml

Store: at -20 °C
